# Supplementary material for: Exponential suppression of bit or phase errors with cyclic error correction
Source: Nature. 2021 Jul 14;595(7867):383–7. doi: 10.1038/s41586-021-03588-y (PMC8279951; doi:10.1038/s41586-021-03588-y)
Supplement: Supplementary file 1 — This file contains Supplementary Information, including Supplementary Figures 1-28, Supplementary Tables 1-7, and additional references. [file 41586_2021_3588_MOESM1_ESM.pdf]

nature portfolio

https://doi.org/10.1038/s41586-021-03588-y

Supplementary information

Exponential suppression of bit or phase errors with cyclic error correction

In the format provided by the authors and unedited

Supplementary information for
"Exponential suppression of bit or phase errors with cyclic error correction"

Google AI Quantum
(Dated: April 15, 2021)

CONTENTS

I. Improvements to the Sycamore device 1

II. CZ gate metrics 1

III. Calibrating phase corrections - ADEPT 2

IV. Data for bit flip code 2

V. Logical error probabilities without post-selection 4

VI. The $d = 2$ surface code 5

VII. Quantifying Lambda 7

VIII. Circuit simulations with Pauli noise 9
- A. A Description of a Component-Error Model for Simulations 10
- B. Comparing Component-Error Simulations to the Experiments 10
- C. Error Budgeting: Constructing a Linear Model Relating Component Errors to Inverse of Lambda 12

IX. Probability p_{ij} of error-paired detection events 13
- A. Error graph and correlation matrix p_{ij} 13
- B. Fluctuations of the p_{ij} elements 15
- C. Experimental results for p_{ij} 16

X. Comparison of Edge Weighting Methods for Matching 23

XI. Dynamical decoupling of data qubits 23

XII. Qubit Frequency Optimization 25

XIII. Overview of error correction experiments 26

References 28

I. IMPROVEMENTS TO THE SYCAMORE DEVICE

The Sycamore processor used in this work has a number of improvements over the original demonstration introduced in Ref. [1]. This revision largely focused on optimizing the readout circuit, to reduce readout times and minimize crosstalk during simultaneous measurement. In particular, the resonator ringdown time $1/\kappa$, resonator-qubit coupling g, and parasitic resonator-resonator coupling were all optimized.

The resonator ringdown times $1/\kappa$ shown in Fig. S1 have a number of important effects on the performance of error correction experiments. The ringdown time sets the timescale for both the depletion of readout photons as well as qubit reset [2]. Both of these operations rely on passively depopulating the resonator, and require waiting a number of ringdown times to bring the resonator photon number to a sufficiently low value ($\tilde{1} \times 10^{-3}$). This time spent waiting is costly since a dominant source of error in our system is decoherence on the data qubits while the measurement and reset operations complete. Thus, fast $1/\kappa$ is a critical processor parameter for error correction with dispersive readout. These improvements to $1/\kappa$ were realized by correcting an impedance mismatch on the control wiring in the interior of the qubit array, which caused larger variations in ringdown times across the chip in the original generation. Additionally, we increased the qubit-resonator coupling g to ensure more-optimal matching of the dispersive shift χ to the new resonator linewidths κ.

While this new Sycamore generation has tighter spread in resonator ringdown times, operation times for QEC are still limited by the slowest measure qubit readout resonator. The specific chain of 21 qubits used in the repetition code was chosen to minimize the longest resonator ringdown time among measure qubits. Future generations of the device will need to further decrease the spread in resonator ringdown time in order to achieve better QEC performance.

Finally, the original Sycamore processor had undesired resonator-resonator couplings across the center of the device, affectionately known as "the bacon strip" within our team and described in Fig. S20 of Ref. [1]. These crosstalk effects heavily constrained simultaneous readout operations and required very specific qubit frequency configurations during readout. These crosstalk effects were minimized in our new revision by increasing the frequency detuning of resonators that are physically close together on the processor, as well as optimizing the geometry of the qubit-resonator coupling capacitor to reduce stray capacitive coupling.

II. CZ GATE METRICS

In Fig. S2, we plot additional metrics which characterize the error rates of our CZ gate. In both sub-figures, we characterize the gate with cross-entropy benchmarking

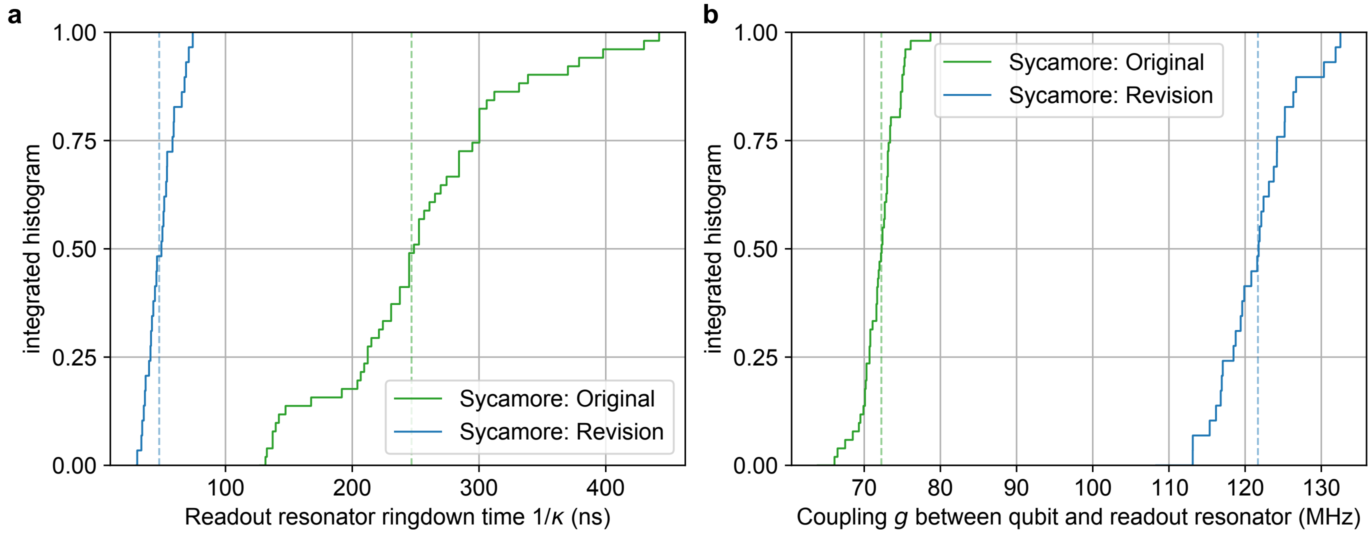

FIG. S1. Readout coupling and ringdown times for Sycamore generations. (Left) Integrated histogram (ECDF) of qubit readout resonator ringdown times $1/\kappa$ across the original (green) and new (blue) generation of the Sycamore processor. The newer generation processor has ringdown times that are approximately 5 times faster, a critical enabler of fast repetitive measurement and reset operations. A focus of future devices will be to tighten this distribution further. (Right) Integrated histogram (ECDF) of coupling between the qubit and readout resonator, where coupling is increased to match larger resonator linewidth.

(XEB), in which we execute circuits composed of varying numbers of cycles, where each cycle consists of random $\pi/2$ rotations on both qubits followed by a CZ gate [1].

III. CALIBRATING PHASE CORRECTIONS - ADEPT

When performing quantum algorithms, the best performance is typically achieved when the calibration procedure is well-matched to the algorithm under test. Here, we introduce single qubit Z phase corrections into the QEC circuit on data and measure qubits as a final calibration step, see Fig. S3. These phase corrections help to compensate for differences between when components are calibrated in isolation and when they are composed in a complex circuit. To calibrate these corrections, we use Active Detection Event Parameter Tuning (ADEPT) as described in Ref. [4], where we choose values that minimize the local detection event fraction.

These Z corrections are virtual and are implemented by updating the frame of subsequent microwave rotations. The origin of these corrections has not been definitively determined, but we believe they arise from a combination of factors including transients from flux pulses, microwave and flux crosstalk, and stray coupling. In addition to lowering the error rate, these corrections also minimize the impact of system drift by keeping qubits first-order insensitive to a variety of phase errors.

Given that these corrections mitigate Z errors, they manifest slightly differently in each experiment. In our error correction circuits, Z errors commute through CZ gates and a single correction can handle corrections for large portions of the circuit. The bit-flip code is insensitive to Z errors on the data qubits, thus we only use corrections on the measure qubits. For the phase-flip code, we use corrections on both the measure and data qubits. For the surface code, we use corrections on the measure qubits and two corrections for each data qubit, which can be understood as correcting for Z errors when the data qubit is in the X basis, and another correction for correcting Z errors when the data qubit is in the Z basis.

IV. DATA FOR BIT FLIP CODE

In addition to the phase flip code that is primarily described in the main text, we also ran a bit flip code for which the logical error rates are shown in Fig. 3c of the main text. The experimental implementation of the bit flip code is similar to the phase flip code except for the following differences:

- Initialization and measurements are performed in the Z basis instead of X.
- The stabilizers used are Z type instead of X type, which means that the the data qubits do not have Hadamards at the beginning and end of each stabilizer round, and parity is measured in the Z basis rather than X.
- We do not run dynamical decoupling pulses on the data qubits during measurement.

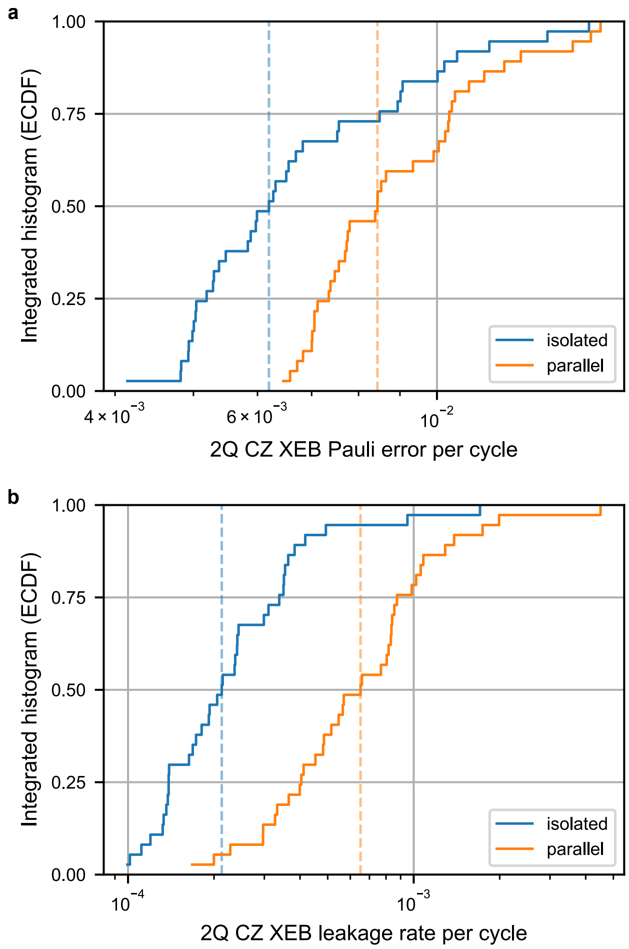

FIG. S2. **a** Typical integrated histogram (ECDF) over pairs of 2-qubit CZ XEB Pauli error rates per cycle, under isolated (blue) and parallel (orange) operation. Note that these errors include two single-qubit gates per cycle, which typically add 2×10^{-3} Pauli error in total. In both cases, we define error with respect to the best-fit single-qubit phases [1]. We attribute the $\tilde{0}.2\%$ increase in error to stray interactions with other qubits when they are excited during parallel operation. **b** Same, but for average leakage rate per cycle, deduced by measuring the $|2\rangle$ population of the higher frequency qubit in each pair vs. number of cycles and fitting to a rate equation as in Ref. [3]. Leakage to the lower qubit as well as leakage from single-qubit gates is $> 10\times$ smaller and not shown. For isolated CZ gates, we attribute roughly half of the leakage to coherent error due to pulse distortion, and the other half due to dephasing, both of which cause an imperfect full swap and back between $|11\rangle$ and $|20\rangle$. As with the total gate error, we attribute the increase in leakage in parallel operation to stray interactions with other qubits.

- Finally, prior to measurement in every round, we flip all of the data qubits with a π pulse to ensure that the data qubits do not collapse into the ground state and remain there, which would artificially reduce logical error probabilities.

In Fig. S4, we show detection fractions and two point

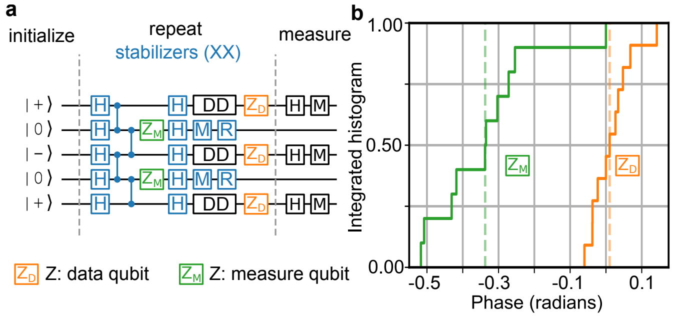

FIG. S3. **Single qubit phase corrections in the phase-flip repetition code circuit. a,** We introduce single qubit variable angle Z corrections into the circuit on the data and measure qubits for the phase-flip repetition code, other codes behave slightly differently, see text. These corrections minimize phase errors that occur between calibrating components individually and when composing algorithms. **b,** Values of the phase corrections. The measure qubit corrections (green) are significant and dominated by transient behavior of the large flux pulses used in the measurement and readout operations. The data qubit corrections (orange) are smaller and centered about zero, but non-negligible.

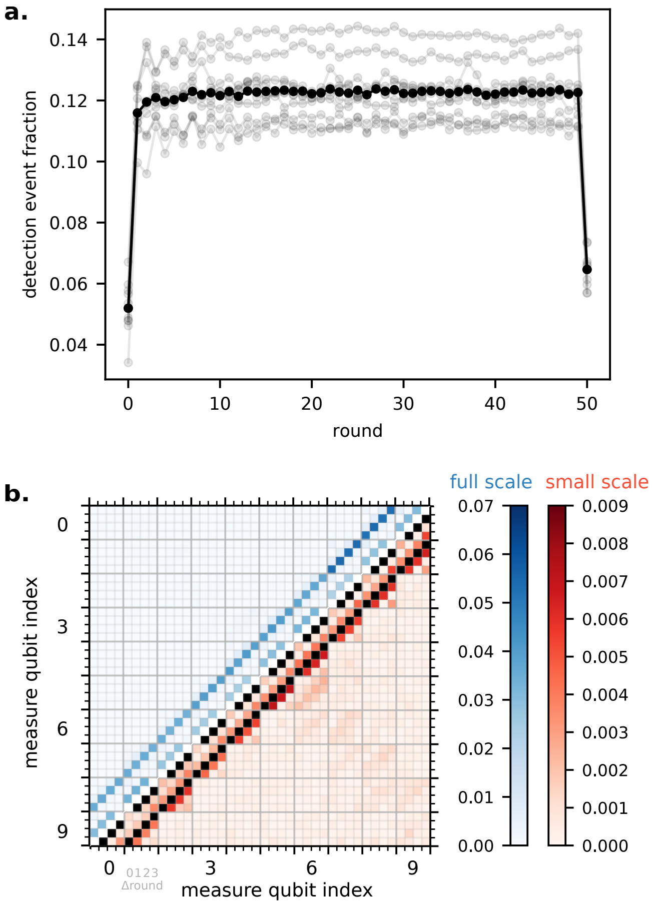

FIG. S4. **a,** Detection event fraction for a 50 round bit flip code, similar to Fig. 1d of the main text. **b,** p_{ij} correlation matrix for the 50 round bit flip code, similar to Fig. 2c of the main text

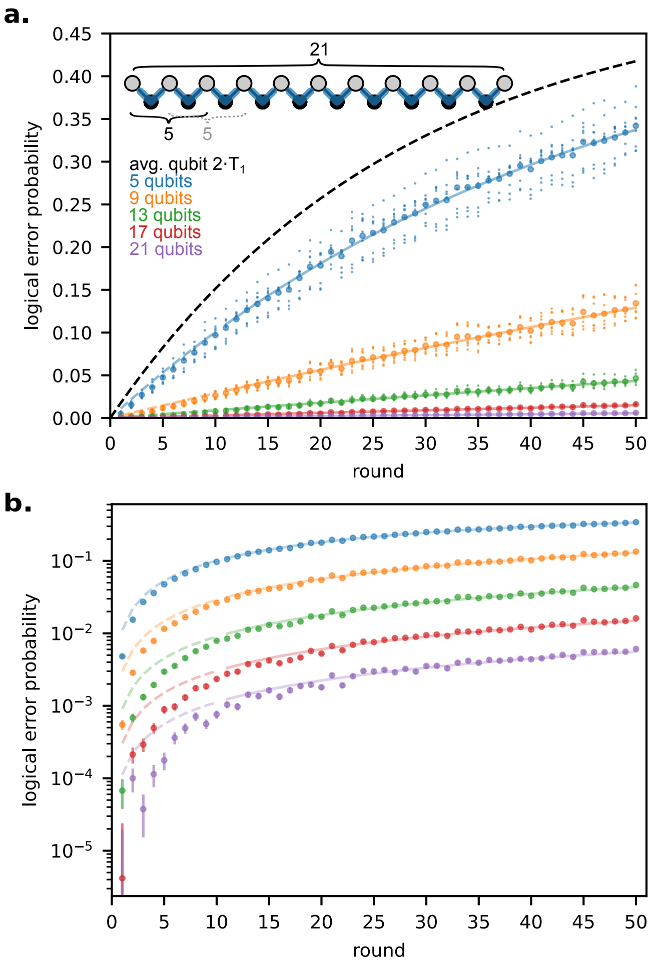

FIG. S5. **a,** Logical error probabilities vs number of detection rounds for the bit flip code, similar to Fig. 3a of the main text. **b,** Semilog plot of logical error probabilities, similar to Fig. 3b of the main text. Lines depict fits to $2P_{\mathrm{error}} = 1-(1-2\epsilon_L)^{n_{\mathrm{rounds}}}$ as in the main text for rounds greater than 10.

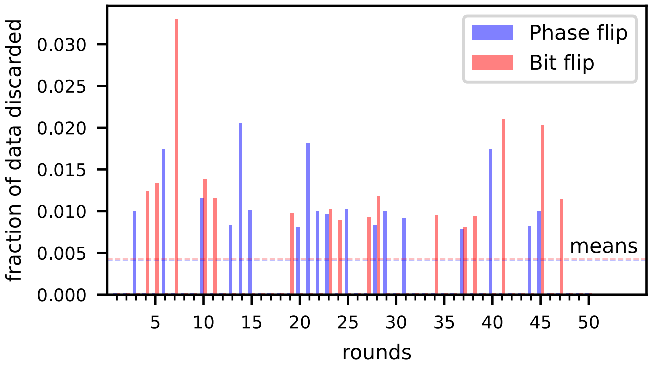

FIG. S6. Fraction of data discarded for each run of the repetition code, for both phase- and bit-flip codes. The dashed lines indicate the mean fraction of data discarded, around 0.4% for both phase and bit-flip codes.

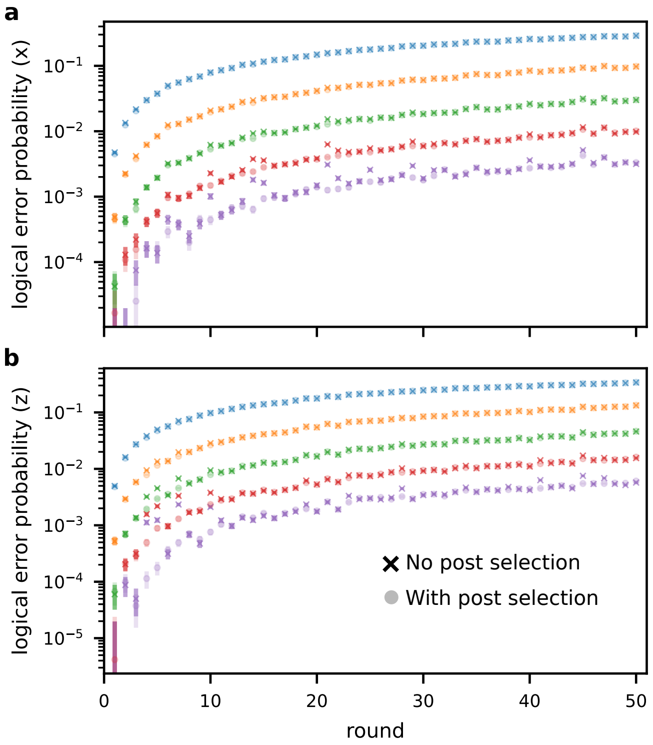

FIG. S7. Logical error probabilities for the **a,** phase flip code and **b,** bit flip code, comparing with (light circles) and without (dark Xs) post-selection of high-energy events.

correlations for the 50 round bit flip code, and in Fig. S5, we show the logical error probabilities for rounds 1-50 of the bit flip code.

V. LOGICAL ERROR PROBABILITIES WITHOUT POST-SELECTION

Logical error probabilities shown in Fig. 3 of the main text were computed while excluding device-wide correlated error events which we attributed to high energy particles. In Fig. S6, we show the fraction of data that was discarded for every number of rounds in the phase and bit flip codes. In Fig. S7, we show the logical error probabilities when high energy events are not discarded, with the post selected logical error probabilities (same as Fig. of the main text) also shown for comparison. To within the uncertainty from fitting, values of Λ_X and Λ_Z do not change when we do not discard data from high energy events.

Additionally, in Fig. S8, we show two p_{ij} matrices derived from the data displayed in Fig. 4d of the main text. The first matrix shown in Fig. S8a is computed from the first 1000 runs of the experiment as a baseline, while the second matrix shown in Fig. S8b is computed from the 1000 runs immediately following the start of the high energy event. We use the same number of runs for both baseline and the high energy event so that the measurement noise floor for the p_{ij} matrix is consistent, which for 1000 runs is about 10 times larger than what is shown in

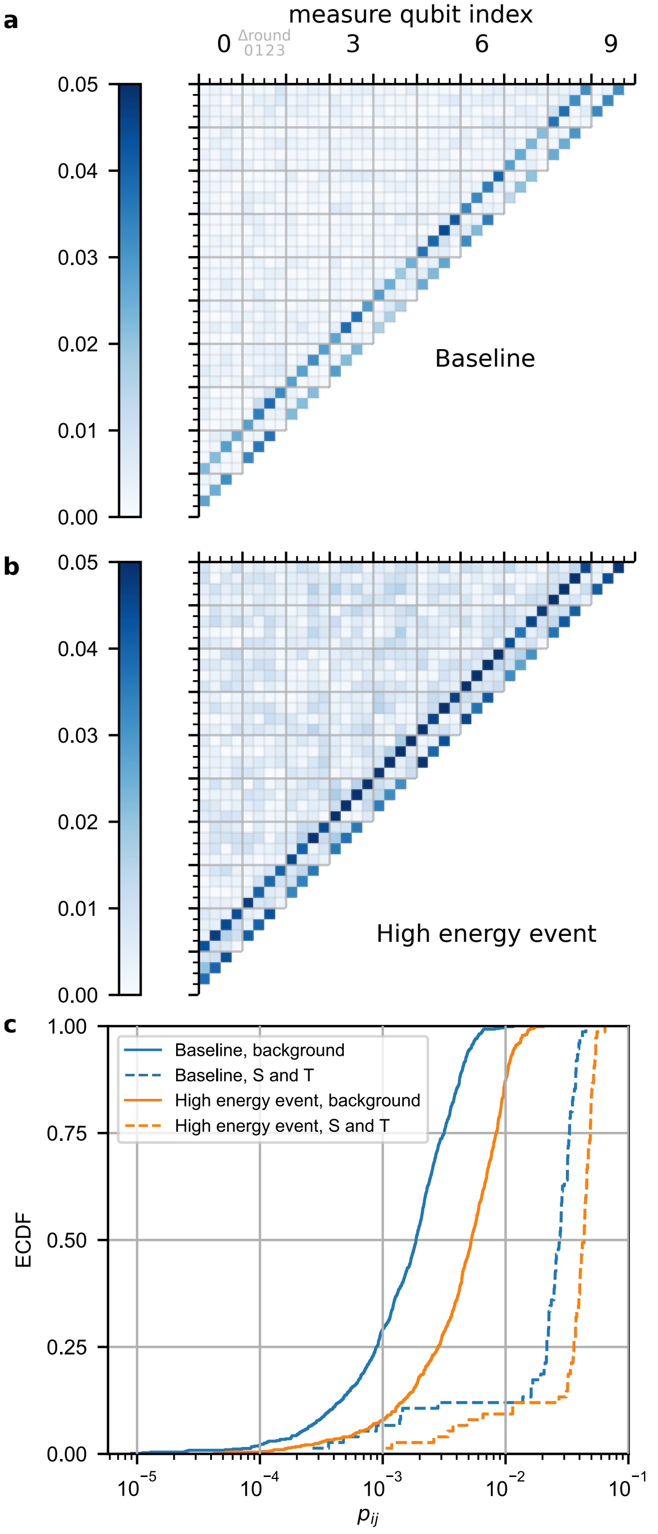

FIG. S8. **a,** Baseline p_{ij} matrix for the first 1000 runs of the data shown in the top of Fig. 4d, where no high energy event is present. As in Fig 2, we average together 4-round segments of the data. **b,** p_{ij} matrix for the 1000 runs immediately following the start of the high energy event in Fig. 4d.c, ECDF of p_{ij} values comparing baseline to high energy event. In the background, meaning matrix elements which are neither space (S) or time (T) edges, we observe a median 0.3% increase in p_{ij} during the high energy event. In S and T elements, we observe a median 1.5% increase.

Fig. 2 of the main text. Finally, Fig. S8c compares the cumulative distributions of the p_{ij} values. The device wide nature of the high energy event breaks the assumption of pairwise correlations used to compute p_{ij}. Nevertheless, we observe a significant increase in the p_{ij} values during the high energy event, both in the values of the expected space and time edges, as well as in the background noise.

VI. THE $d = 2$ SURFACE CODE

We implement a logical qubit in the distance-2 surface code, the smallest non-trivial example of a surface code logical qubit [5, 6]. The physical layout is depicted in Fig. S9a-b, consisting of a 2×2 array of data qubits, indexed 0 to 3, subject to three stabilizer measurements Z_0Z_1, $X_0X_1X_2X_3$, and Z_2Z_3.

Since there are only four data qubits, it is straightforward to write explicit quantum states for the Z_L and X_L eigenstates. Consider the case where the three stabilizer values are all +1. Then, the logical qubit exists in the two-dimensional ground state manifold of the Hamiltonian [7]

$$H = -X_0X_1X_2X_3 - Z_0Z_1 - Z_2Z_3. \qquad (1)$$

We can isolate specific logical states using the logical operators $Z_L = Z_0Z_2$ and $X_L = X_0X_1$ shown in Fig. S9c. For example, $|0_L\rangle$ (+1 eigenstate of Z_L) is the unique ground state of $H - Z_L$. An alternative way to identify $|0_L\rangle$ is to start with $|\psi_0\psi_1\psi_2\psi_3\rangle = |0000\rangle$, which is a +1 eigenstate of Z_L and both Z stabilizers, and then project it into the $X_0X_1X_2X_3 = +1$ subspace with the projection operator $(1 + X_0X_1X_2X_3)/2$. The logical states are

$$\begin{aligned}
|0_L\rangle &= (|0000\rangle + |1111\rangle)/\sqrt{2}\\
|1_L\rangle &= X_L|0_L\rangle = (|0011\rangle + |1100\rangle)/\sqrt{2}\\
|+_L\rangle &= (|0_L\rangle + |1_L\rangle)/\sqrt{2}\\
&= (|0000\rangle + |1111\rangle + |0011\rangle + |1100\rangle)/\sqrt{4}\\
|-_L\rangle &= (|0_L\rangle - |1_L\rangle)/\sqrt{2}\\
&= (|0000\rangle + |1111\rangle - |0011\rangle - |1100\rangle)/\sqrt{4}.
\end{aligned}$$

It is also possible for some stabilizer values to be -1. For example, if $X_0X_1X_2X_3 = -1$ but the others are +1, then we identify $|0_L\rangle = (|0000\rangle - |1111\rangle)/\sqrt{2}$, differing from the +1 case by Z_0 (or any Z_i). Initializing to $|0000\rangle$ and projectively measuring $X_0X_1X_2X_3$, this would be the outcome half the time (also see Fig. S11a).

In our experiments, we explore all 8 stabilizer value combinations, which is representative of stabilizer values that would be encountered by a long-lived logical qubit. In particular, we initialize the data qubits to each of the 16 possible bitstrings, such as $|0111\rangle$. For experiments in the logical Z basis, we proceed directly with stabilizer measurements, and the Z stabilizers and Z_L are already well-defined (for $|0111\rangle$, $Z_0Z_1 = -1$, $Z_2Z_3 = +1$, and

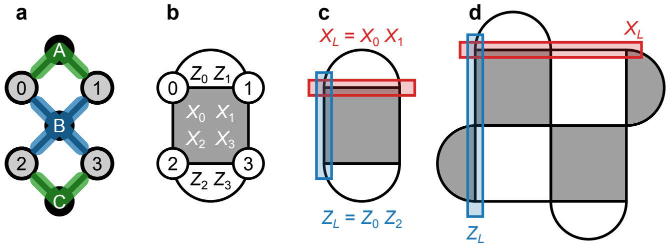

FIG. S9. **Stabilizers and logical operators. a,** Layout of the distance-2 logical qubit as depicted in Fig. 1a, with the data qubits labeled 0, 1, 2, 3, and the measure qubits labeled A, B, C. **b,** The same logical qubit depicted in a more standard lattice surgery surface code notation, as in Ref. [8]. The Z stabilizers are light tiles (Z_0Z_1 and Z_2Z_3), and the X stabilizer is a dark tile ($X_0X_1X_2X_3$). **c,** The logical operators $X_L = X_0X_1$ and $Z_L = Z_0Z_2$, which cross at qubit 0, so $[X_L, Z_L] \neq 0$. **d,** A distance-3 logical qubit and its logical operators, analogous to **c**, with 9 data qubits and 8 stabilizers.

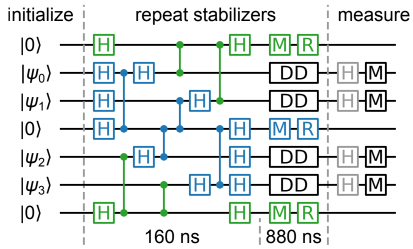

FIG. S10. **Surface code quantum circuit.** Quantum circuit implementing repeated Z (green) and X (blue) stabilizers, analogous to Fig. 1c. The stabilizer circuit is longer (four CZ layers) because of the weight-4 X stabilizer. For X_L logical measurements, we include Hadamard gates on each data qubit prior to measurement, shown in gray; these are omitted for Z_L logical measurements.

$Z_L = -1$). The first $X_0X_1X_2X_3$ measurement is randomly ± 1. For experiments in the logical X basis, we perform Hadamards on all four data qubits before proceeding with the stabilizer measurements, so $|0111\rangle$ becomes $|+---\rangle$. Now the X stabilizer and X_L are well-defined (for $|+---\rangle$, $X_0X_1X_2X_3 = -1$ and $X_L = -1$), and the first Z stabilizer measurements are each randomly ± 1. We show the specific quantum circuit for these experiments, analogous to Fig. 1c, in Fig. S10. Note that the placement of some of the Hadamard gates is flexible. For example, for the top measure qubit shown in Fig. S10, the Hadamard could in principle be placed anywhere prior to the first CZ which is operating on that qubit with no change to the stabilizer. We first rule out placing the Hadamard *during* the CZ gate, as the effect of simultaneously operating single- and two-qubit gates on gate fidelities has not been explored in depth yet. With two possible locations remaining, our software defaulted to placing that Hadamard as early as possible. However, a potentially better choice would have been to defer the Hadamard until immediately before the CZ gate, so that the measure qubit would stay in $|0\rangle$ for longer and be exposed to less decoherence. Since the length of the CZ gate is only 26 ns compared to a mean T_2 of $T_2 = 19\,\mu s$, we expect this oversight to have a negligible effect on the overall results.

Note that to prepare a logical X_L or Z_L eigenstate, it is important to initialize all the data qubits in the same basis (X or Z) as the intended logical qubit state. Then, the data qubit state is an eigenstate of all the stabilizers of the same type as the logical operator, and any errors of the opposite type can be detected in the first round. We show standard Z and X initializations in Fig. S11a-b. Alternatively, consider $|{+}{+}00\rangle$, shown in Fig. S11c, which is employed in Ref. [6]. The first $X_0X_1X_2X_3$ measurement will be random, so no Z errors can be detected on the first round, risking a logical error in X_L. Moreover, although $|{+}{+}00\rangle$ is an eigenstate of $X_L = X_0X_1$, it

TABLE S1. Linear fits for experimental and simulated data of logical error probability vs round in the $d = 2$ surface code. Note that when fitting, we exclude the first data point where only one round of the code is executed because it has an artificially low error rate.

	Slope	Intercept
X experiment	$1.72 \pm 0.10 \times 10^{-3}$	$2.42 \pm 0.20 \times 10^{-3}$
X simulation	$1.48 \pm 0.01 \times 10^{-3}$	$0.31 \pm 0.01 \times 10^{-3}$
Z experiment	$2.48 \pm 0.10 \times 10^{-3}$	$2.18 \pm 0.30 \times 10^{-3}$
Z simulation	$1.58 \pm 0.01 \times 10^{-3}$	$1.40 \pm 0.01 \times 10^{-3}$

is not an eigenstate of $X_L' = (X_0X_1X_2X_3)X_L = X_2X_3$, an equally valid logical operator.

This encoding can detect any single error, but because it is only distance-2, the code cannot be used to correct for errors, as shown in Fig. S11d-e. Any single error on a data qubit leads to an ambiguous syndrome, where it is unclear if a logical operator has been affected. This is distinct from the larger distance-3 logical qubit (see Fig. S9d), where any single error can be corrected unambiguously (distance-d can accommodate any $(d-1)/2$ errors).

Consequently, any time we observe a detection event in a run, we simply discard that run. As we increase the number of rounds, we increase the probability that there has been a detection event, so the fraction of runs we keep decreases exponentially, as shown in Fig. 4c of the main text. Empirically, we remove about 27% of runs each round, which agrees well with simulations of the experiment.

At the end of each run, we measure the data qubits in the basis matching the logical basis of the experiment, either X or Z, and evaluate the appropriate logical operator. We identify a logical error if the logical measurement outcome differs from the value we initialized. By post-selecting only runs without detection events, we avoid most logical errors. However, two simultaneous errors can be undetectable and lead to logical errors, such as X_0X_1, which flips Z_L. Following post-selection, the

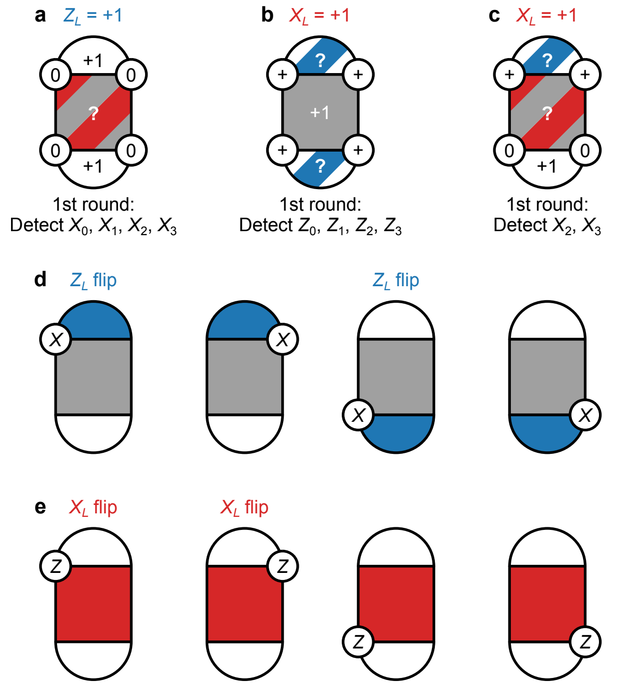

FIG. S11. **Error detection.** **a,** Example initialization to $|0000\rangle$ prior to the first round of stabilizer measurements. This is a +1 eigenstate of Z_L and both Z stabilizers. In the first round, any X error can be detected. However, the first X stabilizer measurement will be random, so no Z errors can be detected. **b,** $|{+}{+}{+}{+}\rangle$ is a +1 eigenstate of X_L and the X stabilizer. In the first round, any Z error can be detected, but the two Z stabilizers will have random values. **c,** $|{+}{+}00\rangle$ is a +1 eigenstate of X_L and the lower Z stabilizer. As in **a**, the first X stabilizer measurement will be random, so no Z errors can be detected, risking a logical error $X_L = -1$. **d,** Illustration of the detected syndrome for one X error. Note X_0 and X_1 have the same syndrome, but X_0 flips Z_L while X_1 does not. X_2 and X_3 are similar. **e,** Illustration of the detected syndrome for one Z error. All four have the same syndrome, but Z_0 and Z_1 flip X_L while Z_2 and Z_3 do not. In **d**-**e**, there is an implicit decoding procedure: for flipped $X_0X_1X_2X_3$, insert Z_0 correction; for flipped Z_0Z_1, insert X_0 correction; and for flipped Z_2Z_3, insert X_2 correction. When this correction is the wrong choice, which happens for about half of error events, we get logical errors.

probability of a logical error is about 0.002 each round, as shown in Fig. 4d, with numerical fits summarized in Table S1. For comparison, in Ref. [6], about 60% of runs are removed each round, and the logical error probability is about 0.03 each round.

Note that in the simulation, the X basis has slightly lower error than the Z basis. One possible explanation is that the two weight-2 Z stabilizers has two opportunities for measurement error versus one for the single weight-4 X stabilizer. The experimental fits similarly show lower errors in the X basis, but with an even greater difference. Understanding this behavior in depth is an active research area, but we enumerate a few possibilities here. First, there may be bias between X and Z errors in the experiment, while the simulation uses depolarizing Pauli noise. Second, compared to the repetition code, the surface code is more vulnerable to drift and other non-Markovian noise, due to longer stabilizer cycles and more possible locations for phase errors in the stabilizer circuit.

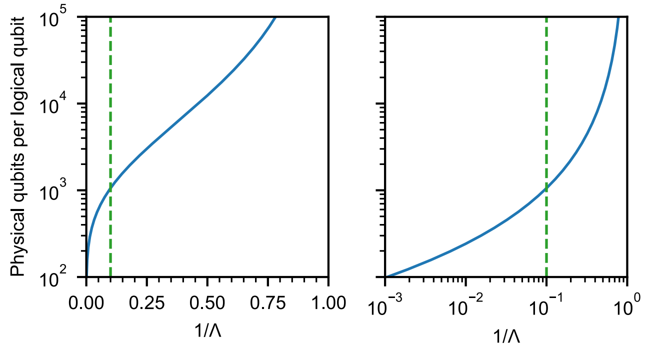

FIG. S12. **Physical qubits per logical qubit.** We estimate the physical qubits required for one logical qubit to achieve an overall logical error suppression of 10^{-12} as a function of the inverse error suppression factor $1/\Lambda$, marking $\Lambda = 10$ with a vertical line. Left: semi-log, right: log-log.

In Fig. 4b, we project the error suppression factor Λ for the surface code. Modest performance improvements will be needed to achieve $\Lambda > 1$, which would be a clear demonstration of operating below threshold error rates, where making the code larger makes it better (even if the absolute error rate is worse than a physical qubit). However, a practical surface code quantum computer would benefit from $\Lambda \sim 10$, which vastly decreases the required physical qubits per logical qubit for a given logical error rate. For example, suppose we want an overall logical error suppression $1/\Lambda^{(d+1)/2} = 10^{-12}$ for a practical computation. For a given Λ, we can solve for distance d and estimate the required number of physical qubits per logical qubit as roughly $2d^2$, as shown in Fig. S12. For $\Lambda = 10$, this corresponds to roughly 1000 physical qubits (distance-23).

VII. QUANTIFYING LAMBDA

Accurately benchmarking the performance of quantum error correction can be confounded by artifacts if experiments are not carefully designed. In particular, boundary effects can introduce different error characteristics that must be understood. Here, we study two types of boundary effects. The first is qubits at code boundaries, which interact with a reduced number of stabilizers and thus participate in a reduced number of entangling gates and may decrease the number of physical errors present. Second, data qubits are subject to less errors in the first round of the code than in the steady-state, and data qubit measurement errors are only relevant in the final round

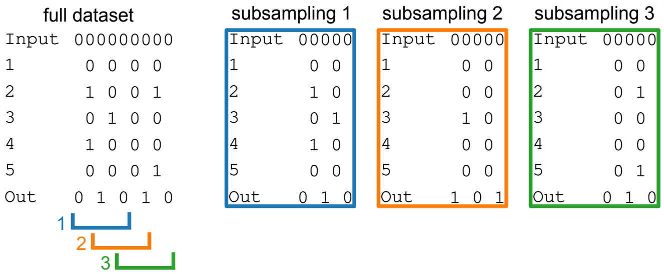

FIG. S13. Example of subsampling a $d = 5$ repetition code dataset into 3 $d = 3$ repetition code datasets.

of measurements.

In our analysis of the repetition code, we use the technique of subsampling outlined in the supplementary materials of [9]. In order to, for example, compare the performance of a d=11 repetition code to a d=3 repetition code, we take a single dataset for the d=11 code, perform matching analysis, then subsample this dataset into a collection of d=3 datasets and perform matching analysis on each sub-dataset. Generally, a repetition code of distance d_s can be subsampled from a larger code of distance d, where $n = d - d_s + 1$ is the number of unique datasets one could produce. This can be understood by considering a line of 9 qubits (for $d = 5$), and uniquely choosing a line of 5 qubits (for $d = 3$) along it, as shown in Fig. S13.

Subsampling has a number of practical advantages. First and foremost, the experimental burden of acquiring data is reduced. In order to quantify the performance of a distance d repetition code as well as all possible configurations of smaller code distances, without subsampling we would need to perform $n_{\text{experiments}} = \sum_{n=1}^{(d-1)/2,\,\text{odd}} d - 2n$. In the case of $d = 11$, subsampling reduces the datasets needed by a factor of 25. Additionally, by using only a single source dataset, we enforce self-consistency in error rates between code distances and reduce sensitivity to systematic errors and system drift that may occur between data acquisition runs. Alternatively, one could collect only a single dataset for each code distance. However, qubits typically have performance variations and the choice of which qubits for which code distance at what time will introduce bias or noise into benchmarking.

In order to understand boundary effects and their impact on repetition code data, we perform simulations using an uncorrelated depolarized Pauli error model. Here, we use a simple error model described by Table S2, where every qubit shares identical error rates. Given these probabilities, we simulate 100,000 runs of a 21 qubit repetition code over 10 QEC rounds.

We process this simulated data to explore the detection event fraction as a function of round, per qubit. We find that the first and last round deviate from the steady-state detection event round, as seen in Fig S14. This discrepancy comes from a difference in circuit structure as well as initial conditions. Before initialization, all qubits begin in the $|0\rangle$ state and suffer no Idling error during the $M + R$ operations that subsequent rounds do. In the last round, the stabilizer outcomes are determined from the final data qubit measurements, and require no data qubit idling or entangling gates. These differences manifest in smaller error rates and thus smaller detection event fractions associated with these rounds.

TABLE S2. Pauli error rates (bit flip error rates for measurement and reset) used in subsequent simulations.

Operation	Error rate
H	1e-3
CZ	5e-3
M	2e-3
R	5e-3
Idle (M + R)	4.4e-2
Idle (H)	7e-4

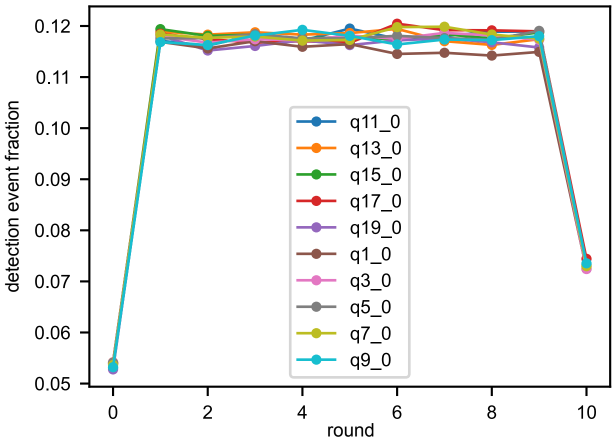

FIG. S14. Simulated repetition code data for 10 QEC rounds and 21 qubits. The plot shows detection event fraction as a function of round. We find a uniform behavior of detection fraction in the intermediate rounds, and different values at the first and last rounds of the code, which differ in circuit structure.

This non-uniformity in detection event fraction must be accounted for when analyzing Λ. In benchmarking QEC, we seek to quantify the logical error rate in the steady-state, but these boundary effects indicate the error rate is slightly different at the beginning and end of the code. Due to this effect, the logical error probabilities will deviate slightly from an exponential decay. To mitigate this behavior, we choose to fit an exponential decay to only experiments with a large number of rounds (greater than 10), where this effect is minimized. This can be seen in Fig. S15, where in this simple model we see logical error probabilities that deviate from an exponential model (dashed, solid lines) at small numbers

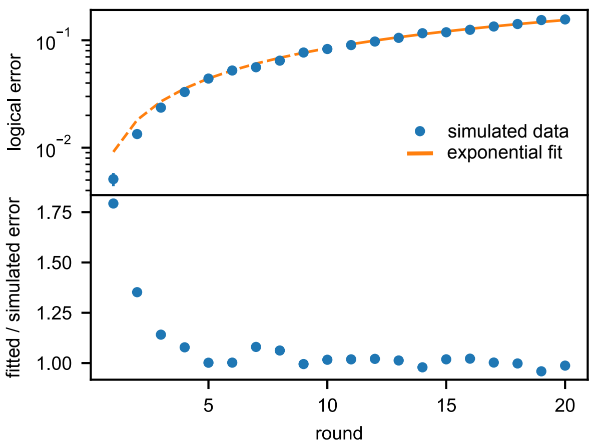

FIG. S15. Logical error probabilities fitted to an exponential model of logical error rate (dashed, solid lines) (distance-3, repetitions = 10,000). At low rounds, we see deviations from the exponential fit due to boundary effects at the start and end of the code, where error rates are reduced as compared to the steady-state of the experiment. This can be seen in the lower graph, where we plot fitted error over simulated error. At low rounds, we find up to nearly a factor of 2 discrepancy. To mitigate this effect, we fit the exponential only to rounds greater than 10. Similar fits can be seen in Fig. 3 of the main text, and in Fig. S5 and Fig. S7.

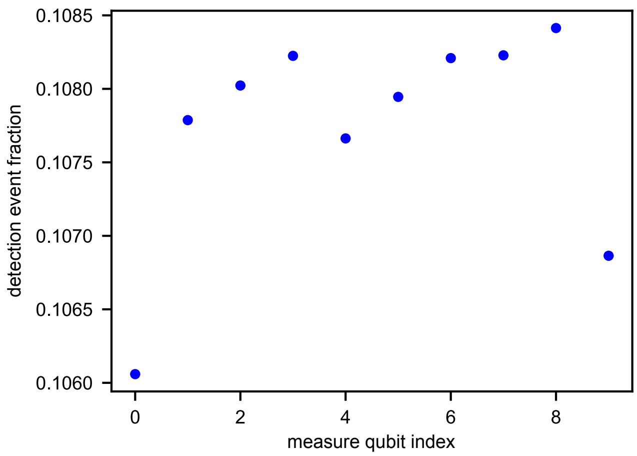

FIG. S16. Detection event fraction vs measure qubit index for a 21 qubit repetition code. Detection event fraction for measure qubits at the edge of the code (index = 0, 9) have lower detection event fraction, as data qubits on the boundary participate in fewer entangling gates.

of rounds. In this regime, the logical error probabilities outperform the steady state and are not predictive of future QEC performance. This discrepancy, here up to a factor of 2, can vary depending on circuit construction and hardware.

In addition to time boundary effects, spatial boundary effects also exist for qubits located at the edge of the code, which participate in less entangling gates. This can be seen in Fig. S16, where the measure qubits at the edges of a simulated 21 qubit repetition code have lower detection event fraction. This introduces a small but systematic difference in comparing subsampled data to experiments that are run in isolation.

VIII. CIRCUIT SIMULATIONS WITH PAULI NOISE

This section describes simulations that approximate errors in the experiment as Pauli errors sampled from probability distributions and inserted into a circuit of Clifford gates. In many quantum error-correcting codes, including repetition codes and surface codes, the bulk of the encoded operations consist only of gates from the Clifford group [10]; the exception is the need to enact logical non-Clifford gates, such as through magic-state distillation [11], which is needed in a fault-tolerant quantum computer but beyond the scope of logical memory experiments like this work. A circuit composed entirely of Clifford gates can be simulated efficiently using the Gottesman-Knill theorem [12], and this description includes noisy circuits where the noise is a probability distribution for randomly inserting a Pauli operator after each gate. Moreover, for stabilizer codes [10], the stabilizers are Pauli operators which can be measured by Clifford gates, so it is convenient to represent errors as a distribution of Pauli errors. We employ this model here — Clifford circuits with Pauli errors — because the simulations can easily scale to modeling large surface codes, such as a distance-23 surface code requiring at least 1057 qubits.

We employ circuit simulations to attempt to understand the relative contributions of errors from different operations, also known as error budgeting. This proceeds in two stages. First, we run simulations of the repetition codes with circuit-noise parameters informed by benchmarking component operations, such as CZ gate error from cross-entropy benchmarking and idling qubit error from measuring T_1 and T_2. We compare the logical error rate in the simulations with the logical errors in the experiment, and see close agreement. We also discuss possible explanations for the gap between experiment and simulation.

Second, we use simulations to estimate the relative contributions of component errors to the logical error rate. We construct an error budget for Λ (see Eqn. (1) of the main text) by attempting to represent its inverse Λ^{-1} as a linear function of the component errors, which we motivate by arguing that Λ^{-1} is approximately linear in the component errors. For such a model, the fraction budgeted to each component is simply given by the weighted contribution of the component error, divided by quantity Λ^{-1}. However, Λ^{-1} is not a perfectly linear function, and we discuss our approach to dealing with this. Our intent with the error budgeting is to determine

what component error rates are necessary to implement a working demonstration of a surface code. We can forecast how a small surface code might perform if run on a device with current error rates, and we can use the error budget to compare tradeoffs in component errors and make design decisions for future devices.

A. A Description of a Component-Error Model for Simulations

We simulate the repetition and surface code experiments in a simplified "circuit noise" model. A circuit is constructed from component operations, including Clifford gates and related operations like initialization or measurement in the eigenbasis of a Pauli operator. A circuit composed of these components can be simulated efficiently, and this set of instructions is sufficient to implement stabilizer codes such as repetition codes and surface codes.

Noise in the circuit is simulated by sampling random Pauli errors and inserting them into the circuit according to the following probability model. For each component, there is a "Pauli error channel," which is a distribution over the possible Pauli errors to insert, including identity for no error (e.g. the distribution has 4 elements for single-qubit operation, or 16 for a two-qubit operation). For each component in the circuit, a Pauli error is sampled according to the distribution associated with that component, and this Pauli operator is inserted after the component. Measurement errors are treated slightly differently, as follows. The binary measurement result is flipped with a probability p, i.e. it goes through a classical binary symmetric channel instead of a Pauli channel. For the circuits used in this work, when a qubit is measured, it is always reset before being used again; this means we do not assume that a measured qubit is left in the state consistent with a measurement result, because we unconditionally reset that qubit before using it again.

The effect of the randomly sampled Pauli errors that are injected into the simulated circuit is to change some of the measurement outcomes from their expected values. For example, an X (bitflip) error that occurs on a data qubit will be detected by the next syndrome circuits that interrogate this data qubit. We collect the syndrome measurements and final data-qubit measurements in the simulation, and process them in the same way as the experiment using minimum-weight matching to infer a most likely location of errors.

Our simulations make some simplifying assumptions about the Pauli error channels. First, we assume that each use of a component of the same type (e.g. every CZ gate) has the same error channel. Of course, it would be straightforward to simulate different error channels for each gate in the circuit. This would also be computationally efficient, but we opt to keep the number of parameters in the simulation relatively small. Second, we further simplify error channels to be parameterized by a single scalar parameter. The error channel for each gate or idle is a depolarizing channel parametrized by a single probability p for any error to occur; for a single-qubit depolarizing channel, each of X, Y, or Z errors has probability $p/3$ to occur; for a two-qubit depolarizing channel, each of the 15 non-identity Paulis has probability $p/15$ to occur. Each reset operation is followed by a quantum bitflip channel (random insertion of Pauli X), and each measurement operation is followed by a classical bitflip channel (random flip of the measurement bit). All components (e.g. every CZ gate) have the same error channel, but different components can have different error probabilities (i.e. measurement error p_{m} can be distinct from the CZ error p_{CZ}).

TABLE S3. Error rates used in bit and phase flip simulations

Component	Bitflip	Phaseflip
DD	5.1e-2	4.1e-2
CZ	6.6e-3	6.6e-3
M	1.9e-2	1.9e-2
R	5.0e-3	5.0e-3
H	1.1e-3	1.1e-3
I	8.4e-4	5.8e-4

There are six types of component operations in our model, which are listed in Tab. S3. Since the error channel on each component has a single parameter, the noise in the simulator has six parameters. We refer to these parameters collectively as a vector denoted x, which we use to relate the component-error probabilities to performance measures of the repetition and surface codes, such as logical-error probability or Λ, the ratio by which logical error improves when code distance is increased by 2.

B. Comparing Component-Error Simulations to the Experiments

To reproduce experimental conditions in the simplified simulator, we try to approximate the error rate in each component with data from benchmarking of those components. The methods for characterizing error are:

- **Single- and two-qubit gates**: cross-entropy benchmarking [13], averaged over the gates used in the experiment. Averages treat one-qubit and two-qubit gates separately.
- **Idle operations**: modeled as memoryless depolarizing channel with decay time constant given the by relevant experiment, meaning "T_1 decay" for the bitflip code and "T_2 decay" for the phaseflip code. T_1 decay means initializing $|1\rangle$ and measuring probability of the state being $|1\rangle$ as a function of time; T_2 decay meanings initializing $|+\rangle$ and measuring decay of this state to the mixed state with

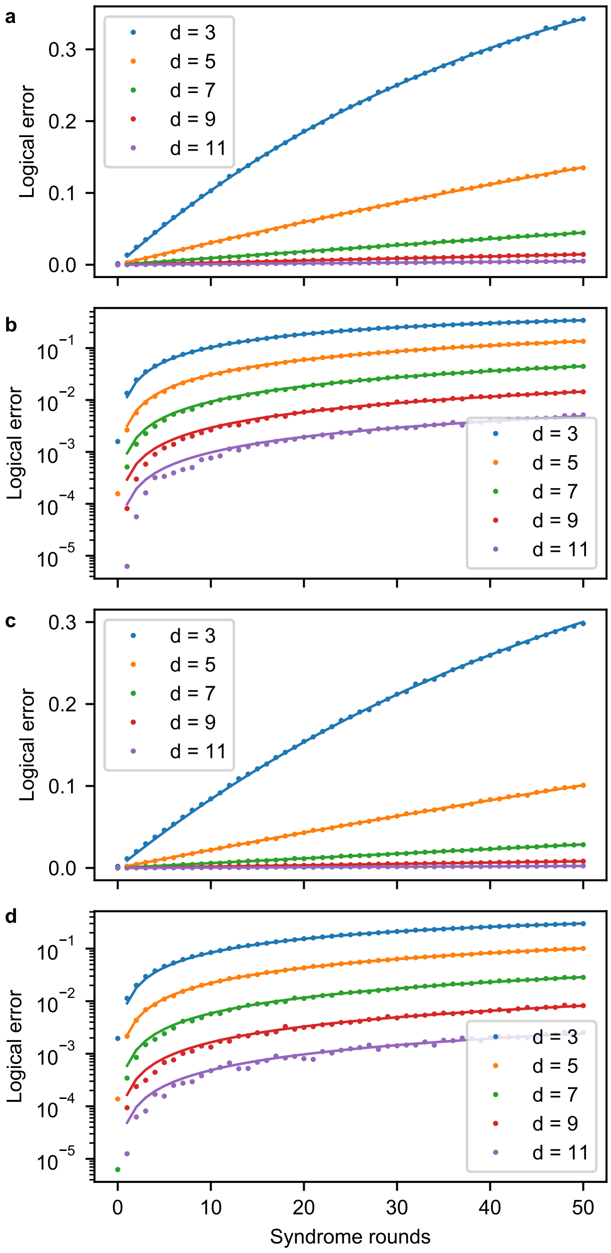

FIG. S17. Simulations of logical-error probability for repetition codes using Pauli-channel noise calibrated to component errors measured in the device. **a,** Logical error vs. number of syndrome rounds for the bit flip code. **b,** Same data as panel **a** (bit flip code), plotted on a log-scaled vertical axis. **c,** Logical error vs. number of syndrome rounds for the phase flip code. **d,** Same data as panel **c** (phase flip code), plotted on a log-scaled vertical axis.

time, while doing CPMG echoing to remove low-frequency phase noise (this dynamical decoupling is also done during idle operations in the phaseflip experiments).

- **Reset and measurement**: These errors are difficult to distinguish; measurement error presents a noise floor for reset characterization. However, for simulation purposes, only the sum of the two error probabilities is important. We characterize reset by performing the reset gate between measurement pulses, preparing the qubit in $|0\rangle$ or $|1\rangle$; the error is the probability of finding $|1\rangle$ after reset. For measurement, we benchmark individual qubits by preparing $|0\rangle$ or $|1\rangle$ and immediately measuring, identifying the error probability. We also benchmark simultaneous readout on all the measure qubits and all the qubits, as in Ref. [1].

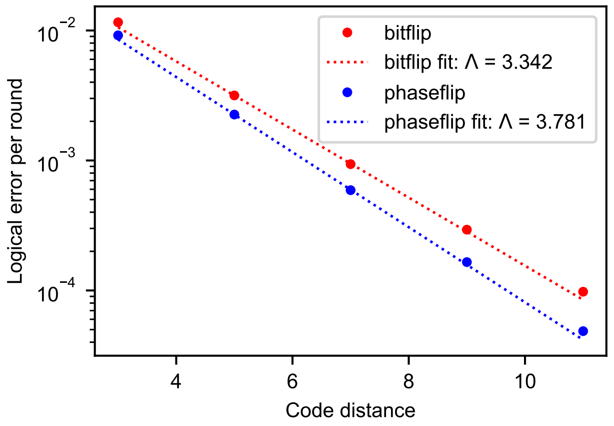

FIG. S18. Logical error vs. code distance for the repetition codes, and a fit to estimate Λ for the two codes.

It is important to note that the model is limited to only simulating Markovian Pauli channels. The associated probability distributions are independent and identically distributed for each type of component. Other important physical effects that we suspect to be present are not included in the model, such as leakage, cross-talk during gates, cosmic rays, parameter drift with time, or any other non-Markovian noise source. The reason for choosing such a limited noise model is that it scales to large problem sizes and allows us to make forecasts of surface codes. Additionally, even crude approximations of effects such as leakage require physical understanding of the origins and dynamics of these errors, and experiments are ongoing in this direction. In future work, we will improve the simulations to incorporate approximations to effects like leakage that are still computationally efficient at large numbers of qubits.

The simulation conditions mirror the experiments in simulating bitflip and phaseflip error-correcting codes with the following parameters. The values of component-error probabilities are those given in the main text, Fig. 4a. The syndrome circuits are executed n_{rounds} times, for n_{rounds} being every integer in the range [1,50]. At each value of n_{rounds}, the simulation is executed $M = 160{,}000$ times. A logical error has occurred if the logical measurement at the end of an error-correction circuit gives

an encoded qubit state different from the initial encoded state. We count the number of simulated logical errors $m_e(n_{\text{rounds}})$ at each value of n_{rounds}, and the logical error probability is calculated as

$$P_{\text{error}}(n_{\text{rounds}}) = m_e(n_{\text{rounds}})/M. \quad (2)$$

For each value of code distance $d \in \{3, 5, 7, 9, 11\}$, we determine the logical error rate $\epsilon_{\text{logical}}$ by fitting

$$P_{\text{error}}(n_{\text{rounds}}) \; = 0.5\left[1 - (1 - 2\epsilon_{\text{logical}})^{n_{\text{rounds}}}\right] \quad (3)$$

to the sampled data. This fitting ansatz has the properties that $P_{\text{error}}(n_{\text{rounds}} = 0) = 0$, it saturates as $P_{\text{error}}(n_{\text{rounds}} \to \infty) = 0.5$, and the error after one round $P_{\text{error}}(1) = \epsilon_{\text{logical}}$. As in the main text, we calculate Λ as the ratio by which logical error improves when increasing the code distance by 2:

$$\Lambda(d) = \epsilon_{\text{logical}}(d)/\epsilon_{\text{logical}}(d+2). \quad (4)$$

The simulated logical error vs. number of syndrome rounds, and fits to this data, are shown in Fig. S17. The simulated logical error rates match well but not perfectly to the experimental results. Figure S18 shows the fitted logical error per round vs. code distance and fits to determine Λ. The error rates are lower, and Λ values are higher, than what is seen in the experiments. We attribute this discrepancy to one of the assumptions of the simulator not holding in experiment. For example, Section IX discusses evidence for cross-talk errors happening during the experiment as well as long time correlations in detection events due to presence of leakage states in the data qubits. Another possibility is that parameter drift during the experiment leads to higher error rates when running error correction than during the component benchmarking that determines the component error probabilities used in the simulation. Said another way, this method of forecasting Λ accounts for about 85% of the error, because it predicts Λ^{-1} values that are about 0.85 of the experimentally measured values, leaving weighted error contributions of about 15% of the total not accounted for. This method was also used to simulate the d=2 surface code, producing the "model" traces in Fig. 4c-d of the main text.

C. Error Budgeting: Constructing a Linear Model Relating Component Errors to Inverse of Lambda

The quantity Λ is used to forecast logical error rate for a quantum code of a given size, so we extend this reasoning to determine what component error rates are needed to realize a target Λ value. We use the convention that Λ is the factor by which logical error is suppressed by increasing code size, where $\Lambda > 1$ means logical error decreases when code size increases. As a ratio, its inverse Λ^{-1} has the same meaning (the factor by which logical error changes when code size increases one step). Moreover, we argue that Λ^{-1} is approximately a linear function of component errors. As in the main text, we say that logical error rate is related to code distance d by $\epsilon_{\text{logical}}(d) \propto \Lambda^{-[(d+1)/2]}$ for d odd. It has been seen in numerical simulations with Pauli-channel noise [14, 15] that for a single physical-error parameter p, $\epsilon_{\text{logical}} \propto (p/p_{\text{th}})^{[(d+1)/2]}$, where p_{th} is the threshold error rate for the chosen code and error model parameterized by p. Hence, a naive comparison of the two approximate expressions would have $\Lambda^{-1} = p/p_{\text{th}}$, meaning that Λ^{-1} is (approximately) linear in p.

For notational simplicity, denote the vector of component error rates as x and let there be a function of component error rates $f(x)$ such that $\Lambda^{-1} = f$. We will assume throughout that $f(0) = 0$, meaning Λ approaches ∞ in the limit errors go to zero. If $f(x)$ were a truly linear function in its arguments, we could calculate the gradient $g = \nabla f$ anywhere to determine f exactly. However, numerical simulations show that this is not the case, and the gradient changes for different choices of the point to linearize around. Since we desire a linear model to form an error budget, we need to make a choice of how to do so; since $f(x)$ is not linear, there is no single "correct" answer.

Our approach is to treat $f(x)$ as if it was a second-order function in its arguments,

$$f(x) \approx gx + 0.5x^{\intercal}Hx, \quad (5)$$

where g is the gradient of f, $(H)_{ij} = \partial^2 f/\partial x_i \partial x_j$ is the Hessian matrix of f, and both are evaluated at $x \to 0^+$. By doing so, we are saying that the second-order terms would capture enough of the nonlinearity in f to provide a good approximation in the domain of interest. We then exploit the following property. For any second-order function f with $f(0) = 0$, there is a linear function given by the first-order Taylor series evaluated at a point $a/2$ such that this linear function coincides with the second-order function at a:

$$\left(\nabla f|_{x=a/2}\right) a = ga + 0.5a^{\intercal}Ha = f(a) \quad (6)$$

To make an error budget for the experimental component-error vector x (values in Fig. 4a of the main text), we use simulations to numerically evaluate the gradient of f at $x/2$, which determines the weights on the error components. From the weights in this linear model, we can produce an estimate of $f = \Lambda^{-1}$ that shows the weighted contribution of each component error. These results are summarized in Tab. S4 and Tab. S5.

We see in these tables that the major source of logical error (more than 50% of the budget) is idling error during the measurement and reset process. This is simply due to T_1 decay times around 15 μs and idle times (880 ns during measurement and reset), leading to an error probability of 4–5% during each such operation. CZ gates and the combined effect of reset and measurement account for most of the remaining errors, with very small contributions from one-qubit gates and idle operations during gates.

TABLE S4. **Error Budget for bit flip code.**

Component	Error rate	Model weight	Contribution to Λ^{-1}	Error-budget percentage
DD	5.1e-2	3.5	0.179	58%
CZ	6.6e-3	11.7	0.077	25%
M	1.9e-2	1.6	0.030	10%
R	5.0e-3	1.6	0.008	3%
H	1.1e-3	3.4	0.004	1%
I	8.4e-4	6.6	0.006	2%

TABLE S5. **Error budget for phase flip code.** *Note that "I" gates are assigned zero weight. The term in the gradient of Λ^{-1} for this component is actually a small negative number that depends on code distance, for example about -1 for Λ between d=3 and d=5. The reason this is negative is that "I" gates only appear on data qubits at the endpoints of the linear chain, and not across the data qubits like the other components. This is why the derivative of Λ^{-1} with respect to "I"-gate probability is negative: errors in this component affect d=3 more than d=5, and the trend continues to higher distances. For the experimentally measured error rate in this component, it has negligible contribution to logical error and hence Λ^{-1}, so we choose to set its weight to zero for the purposes of an error budget.

Component	Error rate	Model weight	Contribution to Λ^{-1}	Error-budget percentage
DD	4.1e-2	3.5	0.144	54%
CZ	6.6e-3	11.9	0.079	29%
M	1.9e-2	1.5	0.029	11%
R	5.0e-3	1.5	0.008	3%
H	1.1e-3	8.0	0.009	3%
I	5.8e-4	0*	0	0%

IX. PROBABILITY p_{ij} OF ERROR-PAIRED DETECTION EVENTS

In this section, we discuss a technique that allows us to characterize error processes in repetition code experiments using correlations between detection events. We refer to this technique as the p_{ij} *correlation matrix method*. We use it to estimate the probability p_{ij} of conventional (e.g., bit or phase flips) and unconventional (e.g., leakage and crosstalk) error processes that produce pairs of detection events at the error graph nodes i and j. We use this technique to produce in-situ diagnostics for QEC operation, and because it extracts detailed error information, it can also inform weights to the decoder.

A. Error graph and correlation matrix p_{ij}

Figure S19 shows an example of the error graph of a quantum bit-flip or phase-flip repetition code. It contains $(N_\mathrm{r}+1)N_\mathrm{mq}$ nodes (vertices), where N_r is the number of rounds $(0, 1, ...N_\mathrm{r} - 1)$ and N_mq is the number of measure qubits (the number of data qubits is then $d = N_\mathrm{mq}+1$, which is also the code distance). Each node i corresponds to readout of a measure qubit (except for the last column of nodes – see below) and can be associated with a pair of error graph coordinates: $i = \{s, t\}$, where $s = 0, 1, ...N_\mathrm{mq} - 1$ is the space-coordinate (mea-

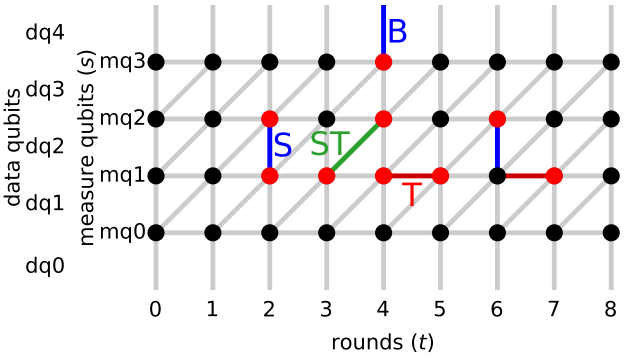

FIG. S19. **Error graph and main edges.** An example of the error graph for $N_\mathrm{mq} = 4$ measure qubits (5 data qubits) and $N_\mathrm{r} = 8$ time rounds. The horizontal axis shows numbering of rounds (t coordinate), the vertical axis shows numbering of measure qubits mq0–mq3 (s coordinate). The dots denote the graph nodes; red dots indicate detection events. The vertical, horizontal and diagonal edges are denoted as Spacelike (S) [including the Boundary (B)], Timelike (T) and Spacetimelike (ST) edges. Positions of data qubits dq0–dq4 (not used in the error graph) are indicated at the left.

sure qubit index) and $t = 0, 1, ...N_\mathrm{r}$ is the time-coordinate (round number). The nodes can also be counted, e.g., in

the "time-first" manner,

$$i = t + (N_\mathrm{r} + 1)s, \tag{7}$$

or in the "space-first" manner,

$$i = s + N_\mathrm{mq}\, t. \tag{8}$$

In each experiment, some of the nodes experience error detection events [9] (or simply "detection events") denoted by red dots in Fig. S19 (black dots denote absence of detection events). By definition, a detection event at node $i = \{s,t\}$ occurs when the corresponding measurement result $m_{\{s,t\}}$ is different from the previous measurement of the same qubit, $x_{\{s,t\}} = m_{\{s,t\}} \oplus m_{\{s,t-1\}}$, where $x_i = 1$ means a detection event at node i, while $x_i = 0$ means no detection event (here $\oplus$ denotes XOR). There are two exceptions to this rule. First, for the column with $t = 0$, instead of non-existing $m_{\{s,-1\}}$ we use the parity of two neighboring data qubits in the initial state (if there is no error, we are supposed to get $x_{\{s,0\}} = 0$). The second special case is for the last column of nodes, $t = N_\mathrm{r}$, which does not correspond to a physical round (physical rounds are $t = 0, 1, ...N_\mathrm{r} - 1$); in this case, instead of non-existing $m_{\{s,N_\mathrm{r}\}}$, we use the parity of neighboring data qubit readouts at the end (after the round $N_\mathrm{r} - 1$), so that $x_{\{s,N_\mathrm{r}\}} = 0$ again indicates the expected no-error situation.

A decoder's task is to use detection events on the error graph to choose one of two given complementary initial states of data qubits (initial parities of neighboring data qubits are given, so the decoder needs to determine only one bit of information). The decoder for this experiment used minimum-weight perfect matching algorithm [9, 14, 16], which connects detection events to each other (pairwise) or to a space-boundary.

In the conventional Pauli error model assumed by the decoder [9], the detection events can be produced only in pairs, corresponding to the edges of the error graph (for the space-boundary edges, only one detection event near the boundary is produced). There are 3 types of such edges – see Fig. S19. Spacelike (S) edges connect nodes $\{s,t\}$ and $\{s+1,t\}$ (the boundary S-edges connect nodes $\{0,t\}$ and $\{N_\mathrm{mq}-1,t\}$ to the corresponding space-boundaries), timelike (T) edges connect nodes $\{s,t\}$ and $\{s,t+1\}$, and spacetimelike (ST, "diagonal") edges connect nodes $\{s,t\}$ and $\{s+1,t+1\}$. In the conventional Pauli error model, a single physical error corresponds to an edge of the error graph.

Note that if two physical errors occur in edges sharing a node (see Fig. S19), then there will be no detection event at this node: two detection events at the same node cancel each other. Therefore it is better to say that a physical error *flips color* (black↔red, $x_i \to 1 - x_i$) of two nodes, instead of producing two detection events.

Now let us discuss how to find the probability p_{ij} of a physical error, which flips colors of both nodes i and j, using experimental statistics of detection events [17]. From experimental data we see that such processes may occur not only when a pair of nodes is connected by a conventional edge on the error graph; therefore, we treat i and j as arbitrary nodes. However, we still assume that such pairs (edges) are uncorrelated with each other. In reality, sometimes there is a correlation between the edges (discussed later); so the assumption of the absence of correlation is a first approximation.

As mentioned above, p_{ij} denotes the probability that two nodes i and j flip color simultaneously. These nodes can also flip color because of other edges connected to i and j separately. However, it is important that these additional flips are independent (uncorrelated) for i and j because they are caused by different physical errors. Therefore, we can consider three uncorrelated processes: node i flips color ($x_i \to 1 - x_i$) with some probability p_i, similarly node j flips color with probability p_j, and both nodes flip color with probability p_{ij}. Since we start with the black color ($x_i = x_j = 0$), the joint probabilities $P(x_i, x_j)$ of detection or no detection events at nodes i and j are

$$P(0,0) = (1 - p_{ij})(1 - p_i)(1 - p_j) + p_{ij}p_ip_j, \tag{9a}$$
$$P(0,1) = (1 - p_{ij})(1 - p_i)p_j + p_{ij}p_i(1 - p_j), \tag{9b}$$
$$P(1,0) = (1 - p_{ij})p_i(1 - p_j) + p_{ij}(1 - p_i)p_j, \tag{9c}$$
$$P(1,1) = (1 - p_{ij})p_ip_j + p_{ij}(1 - p_i)(1 - p_j). \tag{9d}$$

These formulas have obvious meaning, describing combinations of the three processes occurring or not occurring. Note that $P(0,0) + P(0,1) + P(1,0) + P(1,1) = 1$. The relations (9) can also be expressed via the fractions of the detection events (often abbreviated as DEF: detection event fraction) for each node, $\langle x_i\rangle = P(1,0)+P(1,1)$ and $\langle x_j\rangle = P(0,1) + P(1,1)$, and the probability of both detection events, $\langle x_ix_j\rangle = P(1,1)$, which gives

$$\langle x_i\rangle = p_i\,(1 - p_{ij}) + (1 - p_i)\,p_{ij}, \tag{10a}$$
$$\langle x_j\rangle = p_j\,(1 - p_{ij}) + (1 - p_j)\,p_{ij}, \tag{10b}$$
$$\langle x_ix_j\rangle = p_{ij}\,(1 - p_i)\,(1 - p_j) + (1 - p_{ij})\,p_ip_j. \tag{10c}$$

Solving these equations for p_{ij}, p_i, and p_j, we obtain

$$p_{ij} = \frac{1}{2} - \frac{1}{2}\sqrt{1 - \frac{4\,(\langle x_ix_j\rangle - \langle x_i\rangle\langle x_j\rangle)}{1 - 2\langle x_i\rangle - 2\langle x_j\rangle + 4\langle x_ix_j\rangle}}\,, \tag{11}$$

$$p_i = \frac{\langle x_i\rangle - p_{ij}}{1 - 2p_{ij}}\,, \quad p_j = \frac{\langle x_j\rangle - p_{ij}}{1 - 2p_{ij}}\,. \tag{12}$$

We can think about p_{ij} as a symmetric matrix, $p_{ji} = p_{ij}$, with indices corresponding to the nodes ordered either in the "time-first" way (7) or in the "space-first" way (8) – see Figs. S20 and S21 discussed later. Formally, in Eqn. (11) the diagonal elements are the detection fractions, $p_{ii} = \langle x_i\rangle$; however, we usually set them to zero, $p_{ii} \equiv 0$, for clarity of graphical presentation.

Note that in the experimentally relevant case when $p_{ij} \ll 1/4$, Eqn. (11) can be approximated as $(i \neq j)$

$$p_{ij} \approx \frac{\langle x_i x_j\rangle - \langle x_i\rangle\langle x_j\rangle}{(1-2\langle x_i\rangle)(1-2\langle x_j\rangle)}\,. \tag{13}$$

Equation (13) for p_{ij} is Eqn. (2) of the main text. This form shows a clear relation of p_{ij} to the covariance $\langle x_i x_j\rangle - \langle x_i\rangle\langle x_j\rangle$; however, the correction due to the denominator is typically quite significant. For example, for $\langle x_i\rangle \simeq \langle x_j\rangle \simeq 0.11$ (see Fig. 1 of the main text), the denominator in Eqn. (13) is about 0.6. The approximation (13) slightly overestimates Eqn. (11), the correction factor is roughly $(1-3p_{ij})$.

Equation (11) allows us to find accurate individual error probabilities for S, T, and ST edges of the error graph, which are needed for the minimum-weight decoder. However, there is an important exception: the error probability for a boundary S-edge cannot be obtained in this way because it contains only one node. To find the error probability $p_{i\,\rm B}$ for a boundary edge from node i, we use Eqn. (12), $p_{i,\Sigma} = (\langle x_i\rangle - p_{i\,\rm B})/(1-2p_{i\,\rm B})$, in which the "individual flip" probability $p_{i,\Sigma}$ is calculated from already calculated error probabilities for S, T, and ST edges connected to the node i. We essentially sum up the known error probabilities of the connected edges and find the missing error probability (due to the boundary edge) to bring the sum to the DEF $\langle x_i\rangle$. Note, however, that it is not a simple sum of the probabilities because of the "color flipping" procedure, so that the errors p_{ij_1}, p_{ij_2}, ... p_{ij_k} due to k connected edges produce the total flip probability

$$p_{i,\Sigma} = g(p_{ij_k}, ...\, g(p_{ij_3}, g(p_{ij_2}, p_{ij_1}))...), \tag{14}$$
$$g(p,q) \equiv p(1-q) + (1-p)q = p+q-2pq. \tag{15}$$

Thus, after finding $p_{i,\Sigma}$, we calculate the boundary S-edge probability as

$$p_{i\,\rm B} = \frac{\langle x_i\rangle - p_{i,\Sigma}}{1-2p_{i,\Sigma}}\,. \tag{16}$$

Note that this procedure for boundary edges assumes that error processes corresponding to different edges are uncorrelated. In reality this is not a very good assumption (this is why we are actually using a slightly different procedure for boundary edges). A natural way to estimate the effect of correlation between the edges is to use Eqn. (14) for a node i not close to a boundary, summing up the contributions from all connected edges and then comparing the result with the DEF $\langle x_i\rangle$. Doing this test for the phase-flip experiment, we typically find a relative inaccuracy of about 4% (median value), which indicates a reasonably small but still nonzero correlation between the main edges (for the bit-flip experiment the median relative inaccuracy is about 9%). A natural way of thinking about positive correlations between the edges is to assume that some error processes flip color of 4, 6, ... nodes on the error graph, so that the same process increases p_{ij} for several pairs of nodes (this also produces unconventional edges on the error graph reported by p_{ij}). To study correlations between edges, we have generalized the method of p_{ij} to 3-point and 4-point correlators (essentially the "hyperedges"), extending the approach of Eq. (9) to account for more nodes and more error processes. This generalization will be described in a future publication.

B. Fluctuations of the p_{ij} elements

When evaluating Eqn. (11) using experimental data, the p_{ij} values exhibit statistical fluctuations because the averages $\langle x_i x_j\rangle$, $\langle x_i\rangle$, and $\langle x_j\rangle$ are estimated from a large but finite number $N_{\rm expt}$ of experimental realizations (typical values of $N_{\rm expt}$ are between 10^3 and 10^5). In this section we estimate the standard deviation $\sigma_{p_{ij}}$ of statistical fluctuations of the p_{ij} elements.

For the estimate, let us use the approximation (13) and assume the usual experimental case when $\langle x_i\rangle \ll 1$, $\langle x_j\rangle \ll 1$, and $p_{ij} \ll 1$. Then the effect of the denominator fluctuations is negligible in comparison with fluctuations of the numerator (covariance C_{ij}), so

$$\sigma_{p_{ij}} \approx \frac{\sigma_{C_{ij}}}{(1-2\langle x_i\rangle)(1-2\langle x_j\rangle)}\,, \quad C_{ij} = \langle x_i x_j\rangle - \langle x_i\rangle\langle x_j\rangle. \tag{17}$$

Using the form $C_{ij} = \langle (x_i - \langle x_i\rangle)(x_j - \langle x_j\rangle)\rangle$ and using in it true averages $\langle x_i\rangle$ and $\langle x_j\rangle$ instead of averages over $N_{\rm expt}$ realizations (the effect of the change is negligible), we find

$$\sigma_{C_{ij}} = \sqrt{{\rm Var}[(x_i - \langle x_i\rangle)(x_j - \langle x_j\rangle)]/N_{\rm expt}}\,. \tag{18}$$

The variance here is $\langle (x_i - \langle x_i\rangle)^2 (x_j - \langle x_j\rangle)^2\rangle - C_{ij}^2$, in which the first term can be rewritten after some algebra as $C_{ij}(1-2\langle x_i\rangle)(1-2\langle x_j\rangle) + \langle x_i\rangle\langle x_j\rangle(1-\langle x_i\rangle)(1-\langle x_j\rangle)$, using the properties $x_i^2 = x_i$ and $x_j^2 = x_j$. Inserting this form into Eqn. (17) and using $C_{ij}/[(1-2\langle x_i\rangle)(1-2\langle x_j\rangle)] \approx p_{ij}$, we obtain

$$\sigma_{p_{ij}} \approx \frac{\sqrt{p_{ij}(1-p_{ij}) + \dfrac{\langle x_i\rangle\langle x_j\rangle(1-\langle x_i\rangle)(1-\langle x_j\rangle)}{(1-2\langle x_i\rangle)^2(1-2\langle x_j\rangle)^2}}}{\sqrt{N_{\rm expt}}}\,. \tag{19}$$

Note that the first and second terms in the numerator of Eqn. (19) have a clear meaning and can be obtained separately. When p_{ij} is well above the statistical noise floor, $\sigma_{p_{ij}}$ mainly comes from fluctuation of the number of realizations, in which the edge error (color flipping event) has occurred: $N_{\rm expt}p_{ij} \pm \sqrt{N_{\rm expt}p_{ij}(1-p_{ij})}$, as follows from the binomial statistics. It is easy to see that this leads to the first term in Eqn. (19). The second term is the noise floor, coming from the fluctuations of $\langle x_i\rangle$, $\langle x_j\rangle$, and $\langle x_i x_j\rangle$ when $p_{ij}=0$. It can be obtained, e.g., by considering the number of realizations with $x_i = 1$: $N_{x_i=1} = N_{\rm expt}\langle x_i\rangle \pm \sqrt{N_{\rm expt}\langle x_i\rangle(1-\langle x_i\rangle)}$, number of

realizations with $x_i = x_j = 1$: $N_{x_i=x_j=1} = N_{x_i=1}\langle x_j\rangle \pm \sqrt{N_{x_i=1}\langle x_j\rangle(1-\langle x_j\rangle)}$ (with uncorrelated $\pm$), and realizations with $x_i = 0$ and $x_j = 1$: $N_{x_i=0,\,x_j=1} = (N_{\rm expt} - N_{x_i=1})\langle x_j\rangle \pm \sqrt{(N_{\rm expt} - N_{x_i=1})\langle x_j\rangle(1-\langle x_j\rangle)}$ (also with uncorrelated $\pm$). Then calculating the apparent value of the covariance C_{ij} and using it in Eqn. (17), we obtain the noise floor, which gives the second term in Eqn. (19).

As a final simplification, let us neglect the factors $(1-p_{ij})$ and $(1-\langle x_i\rangle)(1-\langle x_j\rangle)$ in Eqn. (19) (this slightly increases $\sigma_{p_{ij}}$, so we are on the safe side), thus obtaining

$$\sigma_{p_{ij}} \approx \frac{1}{\sqrt{N_{\rm expt}}}\sqrt{p_{ij} + \frac{\langle x_i\rangle\langle x_j\rangle}{(1-2\langle x_i\rangle)^2(1-2\langle x_j\rangle)^2}}\,. \quad (20)$$

In our repetition phase-flip code experiments, we have $N_{\rm expt} = 76,000$ realizations and the detection error fractions are $\langle x_i\rangle \simeq \langle x_i\rangle \simeq 0.11$ (slightly bigger, $\simeq 0.12$ in the bit-flip experiments). Thus, the standard deviation of the experimental p_{ij} values that are nominally zero (noise floor) is roughly

$$\sigma_{p_{ij}} \simeq 6\times 10^{-4}. \quad (21)$$

In particular, this is the noise floor seen in the p_{ij} matrix plots shown in Figs. S20 and S21. Additional averaging over the rounds leads to even smaller noise floor ($< 2\times 10^{-4}$) in Fig. 2(c) of the main text.

C. Experimental results for p_{ij}

Figure S20 shows the correlation matrix p_{ij} for a phase-flip code experiment with 21 qubits ($N_{\rm mq} = 10$ measure qubits and 11 data qubits) and $N_{\rm r} = 30$ rounds. In this particular experiment, no cosmic rays events were detected, so no data was discarded from $N_{\rm expt} = 76,000$ runs. The error graph nodes i and j are ordered in the "time-first" way given by Eqn. (7). Figure S20 contains 310×310 pixels, with the color of each pixel determined by the value of the corresponding p_{ij} element. Each axis contains $N_{\rm mq} = 10$ blocks (see grid lines) corresponding to 10 measure qubits indicated on the axes; each block contains $N_{\rm r} + 1 = 31$ points (small ticks on the axes) corresponding to time rounds.

We see that most pixels in Fig. S20 (which are away from the features discussed below) have values close to zero. The fluctuations are consistent with the expected noise floor given by Eqn. (21). The figure is symmetric across the main diagonal (which runs bottom-left to top-right) because $p_{ji} = p_{ij}$. The values on the main diagonal are set to zero.

The most visible features are 4 diagonal lines (2 from each side of the main diagonal), which correspond to S and T edges of the error graph: the T-edge line contains pixels next to the main diagonal, while S-edge line is $N_{\rm r}+1$ pixels away from the main diagonal. The color scale for S and T lines is saturated because the values of p_{ij} for these lines are around 0.03; they are shown in Fig. S22 discussed in more detail below. There is also a less visible line in Fig. S20 next to the S-line (one pixel farther, $N_{\rm r}+2$, from the main diagonal), which corresponds to ST edges. The typical values of p_{ij} for the ST-line are around 0.004. Another well-visible feature in Fig. S20 is a reddish "dirt" near S and T lines for qubits mq1 and mq2 and to a less extent for some other qubits; we attribute this feature to leakage to state $|2\rangle$ in a data qubit. One more feature is short lines ("scars") parallel to the main diagonal, which we attribute to crosstalk. The leakage and crosstalk are discussed later.

S, T, and ST edges. In the conventional theory of the repetition QEC code, the errors are associated only with S, T, and ST edges. The elements of p_{ij} show the probabilities of these errors individually for each edge on the error graph. We emphasize that these probabilities are obtained *in situ*, during the actual operation of the code, in contrast to estimates based on qubit coherence and gate fidelities.

As expected from the conventional theory, S, T, and ST edges are the main features in Fig. S20. The values of p_{ij} elements for these edges are shown in Fig. S22 by blue markers for S-edges, red markers for T-edges, and green markers for ST-edges; the lines are a guide for the eye. The S-edge error probabilities for the boundary edges (denoted dq0 and dq10 in Fig. S22) are calculated using Eqs. (14)–(16); we see that their values are consistent with other S-edges. Each block of blue markers corresponds to a particular data qubit (indicated at the top), markers within a block correspond to time rounds (from 0 to 30, see the horizontal axis). Note that S-edge probabilities for rounds $t = 0$ and $t = 30$ are significantly smaller than for other rounds (emphasizing the need of many rounds in an experiment). This is because S-edge errors in our phase-flip code are mainly due to dephasing of data qubits during readout and reset (or due to energy relaxation for a bit-flip code), while the special rounds $t = 0$ and $t = N_{\rm r}$ do not have these parts of the cycle. For other rounds, the error probability p_{ij} can be crudely estimated as $\tau/2T_2$, where τ is the readout-and-reset time (expected contribution from CZ gates is significantly smaller). In our experiment, $\tau = 0.88\,\mu$s and on average $T_2 \simeq 16\,\mu$s, which gives $\tau/2T_2 \simeq 0.028$. We see that p_{ij} values for S edges (blue symbols) are close to this estimate, though they are different for different data qubits, mostly reflecting variation in T_2 times and also having contributions from gate errors. The integrated histogram for the S-edges is shown by the blue line in the left panel of Fig. S23; the median p_{ij} value is

$$p_{ij}^{\rm S-edge,\, median} \approx 3.0\times 10^{-2}. \quad (22)$$

The T-edge errors (red symbols in Fig. S22) are grouped in blocks corresponding to measure qubits indicated below the red symbols. The T-edge errors are expected to come mainly from the readout errors, but there are also contributions from the gate errors and reset error. Our median readout error is around 0.018;

however, the p_{ij} values are considerably higher, with the median value (see the integrated histogram in Fig. S23) of

$$p_{ij}^{\mathrm{T-edge,\,median}} \approx 2.7 \times 10^{-2}. \quad (23)$$

The error probabilities for ST edges (green symbols in Fig. S22) are much lower than for S or T edges; they are supposed to come mainly from CZ gate errors. The integrated histogram in Fig. S23 (green line) shows for ST edges the median value of

$$p_{ij}^{\mathrm{ST-edge,\,median}} \approx 3.7 \times 10^{-3}. \quad (24)$$

Unconventional edges. Figure S20 clearly shows that in contrast to what is expected from the conventional QEC theory, some correlations between the detection events correspond to error graph edges different from the S, T, and ST types. In particular, there are significantly non-zero p_{ij} values near the lines corresponding to T and S edges, separated from them by a few rounds. The integrated histogram for some types of these edges is shown in the right panel of Fig. S23). As illustrated by the inset, with ST′ we denote the "diagonal" edges similar to the ST edges, but going into the other direction. With 2T, 3T, etc. we denote the edges spanning 2, 3, etc. rounds for the same measure qubit. We see that out of the unconventional edges, 2T edges have the highest typical probability (the median of 1.7×10^{-3}), which is still more than twice smaller than the typical ST-edge probability. A relatively small probability of unconventional edges indicates a high quality of the experiment. Note that before the qubit reset [2] was implemented, the unconventional-edge probabilities were much higher, with 2T probabilities exceeding ST probabilities.

The negative values of p_{ij} for a small fraction of unconventional edges shown in Fig. S23 are consistent with the statistical noise level (21). Note, however, that in some cases, for example, for 2T edges in a high-quality bit-flip experiment, the p_{ij} values can actually be slightly negative. This can be understood using Eqn. (13) as a negative correlation. Indeed, a negative correlation between the nodes can be caused by a negative correlation between the edges. An example is the second-order anticorrelation due to data qubit energy relaxation (an energy relaxation event cannot be immediately followed by another relaxation event), which may cause slightly negative p_{ij} in a bit-flip repetition code experiment [2].

Figure S21 shows the same data as Fig. S20 but with the different ordering of nodes: here we use the "space-first" ordering from Eqn. (8). Then each axis contains $N_{\mathrm{r}} + 1 = 31$ blocks corresponding to time rounds (grid lines), while $N_{\mathrm{mq}} = 10$ points within each block correspond to measure qubits. The S-edges are next to the main diagonal, the T-edges are the diagonal lines separated by 10 pixels from the main diagonal, and the ST edges are on the next diagonal line (11 pixels from the main diagonal). The parallel lines in Fig. S20 separated by 20, 30, etc. pixels from the main diagonal correspond to 2T, 3T, etc. edges. The figure clearly shows that temporal correlations can survive for over 5 rounds.

Leakage to state $|2\rangle$. We attribute the detection-event correlations lasting for several rounds, as seen in Fig. S21, to the leakage to state $|2\rangle$ in data qubits. The same effect causes the "dirt" in Fig. S20 close to S and T lines, with the magnitude of the correlations for several edge types shown in the right panel of Fig. S23. Note that measure qubits are reset to $|0\rangle$ at every round, so non-computational states can survive only in data qubits. For a typical qubit energy relaxation time of $T_1 \simeq 15\,\mu$s and the round duration of 960 μs, we would expect that state $|2\rangle$ should survive on a data qubit for about 8 rounds. Examining Figs. S23 and S21, we see that this estimate is in the right ballpark, but the actual decay of the state $|2\rangle$ can be significantly faster due to hopping of leakage, a subject of ongoing research.

We have found that the amount of leakage is sensitive to minor experimental details. The p_{ij} technique can be used for a fast diagnostic to estimate the level of leakage and to find which qubits suffer a bigger leakage. Specialized experiments have shown [2] that a typical probability of state $|2\rangle$ in a data qubit is around 4×10^{-3}. This magnitude is consistent with the values we extract from the p_{ij} analysis. While this analysis is somewhat involved, we note that ST′ edges have a somewhat similar (though smaller) p_{ij} values due to leakage. For our phase-flip code experiment, the median value for ST′-edge errors is 1.3×10^{-3}, while the biggest value (averaged over rounds) is 3.3×10^{-3} for data qubit dq2 (as can be seen from Fig. S20, dq2 has the biggest leakage). So, as a crude proxy for leakage, we can use

$$p_{ij}^{\mathrm{ST'-edge\,(leakage)}} \lesssim 3 \times 10^{-3}. \quad (25)$$

The 2T edges can also be used to estimate leakage; the biggest 2T-edge value (averaged over rounds) is 3.6×10^{-3} for measure qubit mq2. (All these values are for the phase-flip code; for a bit-flip code there is an additional contribution from "odd-even correlations" due to energy relaxation of data qubits).

Note that during several rounds while a data qubit is in state $|2\rangle$, there is a relatively high probability of detection events at the neighboring measure qubits [2]. This leads to a significant correlation between S-edges (and also T-edges), which negatively affects performance of the minimum-weight-matching decoder. This is why leakage is dangerous for quantum error correction even for a relatively low leakage probability.

Crosstalk features. Short parallel lines ("scar" features) in Fig. S20 far away from the main diagonal indicate the presence of correlations between detection events at qubits, which are far apart along the 1D line of qubits used in the experiment. However, they are actually close to each other on the Sycamore chip – see the top panel of Fig. S24, which shows 10 pairs of measure qubits (indicated by arrows), for which there are visible scars in Fig. S20. We attribute these scar features to the crosstalk.

The lower panel of Fig. S24 shows the values of same-round p_{ij} elements averaged over the rounds, for all pairs of measure qubits except nearest neighbors. While most values are within the statistical noise level, the elements corresponding to the scar features are significantly above the noise floor (bigger values are indicated by orange and green cells). We see that the magnitude of the crosstalk correlations is

$$p_{ij}^{\text{crosstalk}} \lesssim 2 \times 10^{-3}. \tag{26}$$

For the crosstalk pairs shifted in time by one round we find crudely twice smaller edge probabilities.

The long-range correlation between detection events caused by crosstalk are dangerous to the code operation because they can effectively reduce the code distance. However, we see that in our device the crosstalk is quite small and, most importantly, local in physical distance on the chip. Therefore, we expect that in the future it will not present a serious problem in a surface code operation.

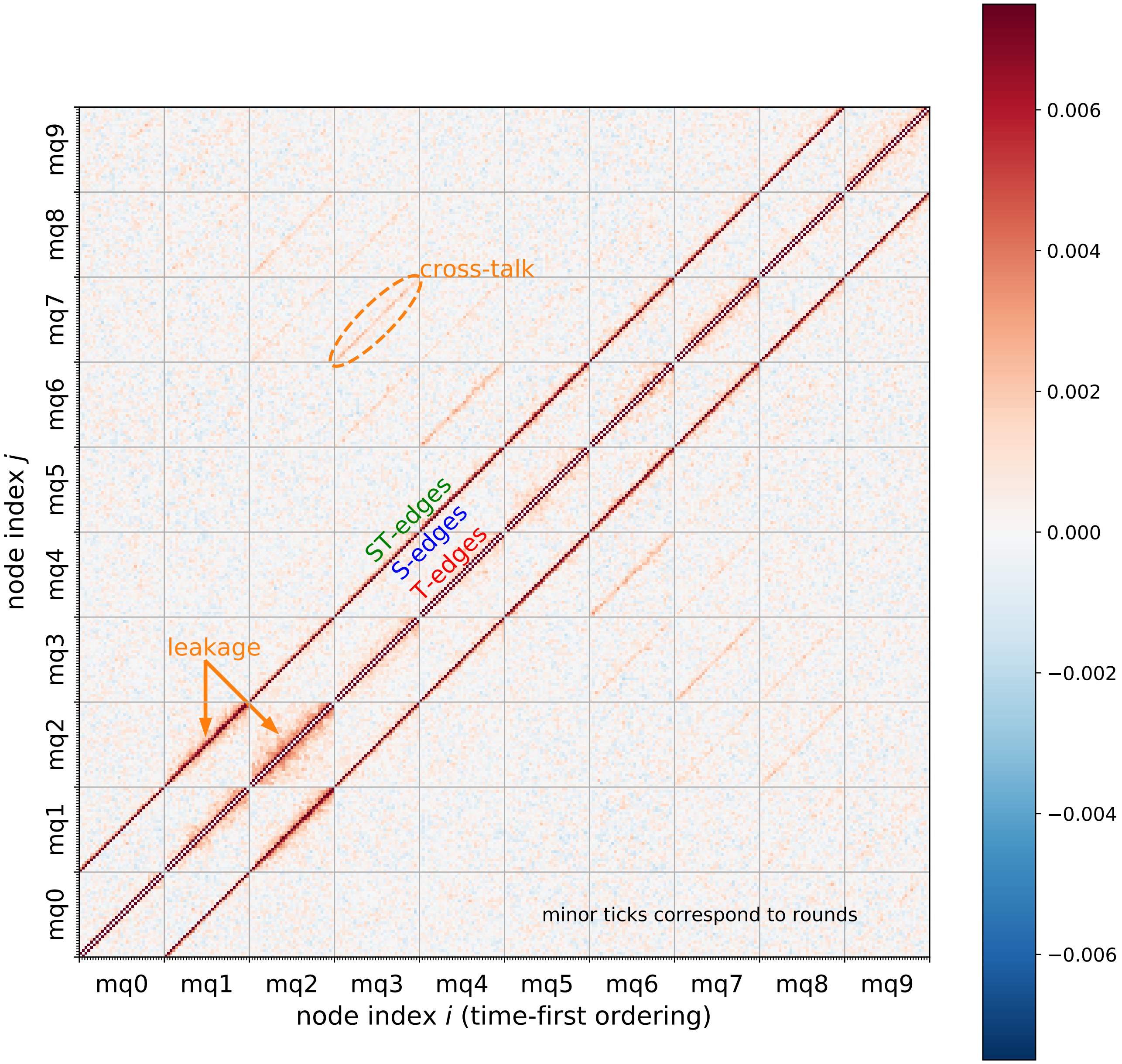

FIG. S20. **Correlation matrix p_{ij}.** A graphical representation of the 310×310 symmetric matrix p_{ij} [Eqn. (11)] for a phase-flip repetition code experiment with $N_{\rm mq} = 10$ measure qubits (11 data qubits) and $N_{\rm r} = 30$ rounds. The color of each pixel depicts the probability p_{ij} for an error process involving error graph nodes i and j. The nodes are ordered in the "time-first" fashion, Eqn. (7), with 10 blocks (separated by grid lines) corresponding to measure qubits (mq0, mq1, ... mq9) and 31 ticks within each block corresponding to time rounds (from $t = 0$ to $t = N_{\rm r}$). The main features are the diagonal lines corresponding to T, S, and ST edges, which are shifted from the main diagonal by 1, 31, and 32 pixels, respectively (ST line is more faint than T and S lines). Additional features are reddish ("dirty") patches near S and T lines, which are due to leakage to state $|2\rangle$ in data qubits, and also short parallel lines ("scars") due to crosstalk. Note that the color bar ranges to 0.007, while probabilities for S and T edges are above this truncation.

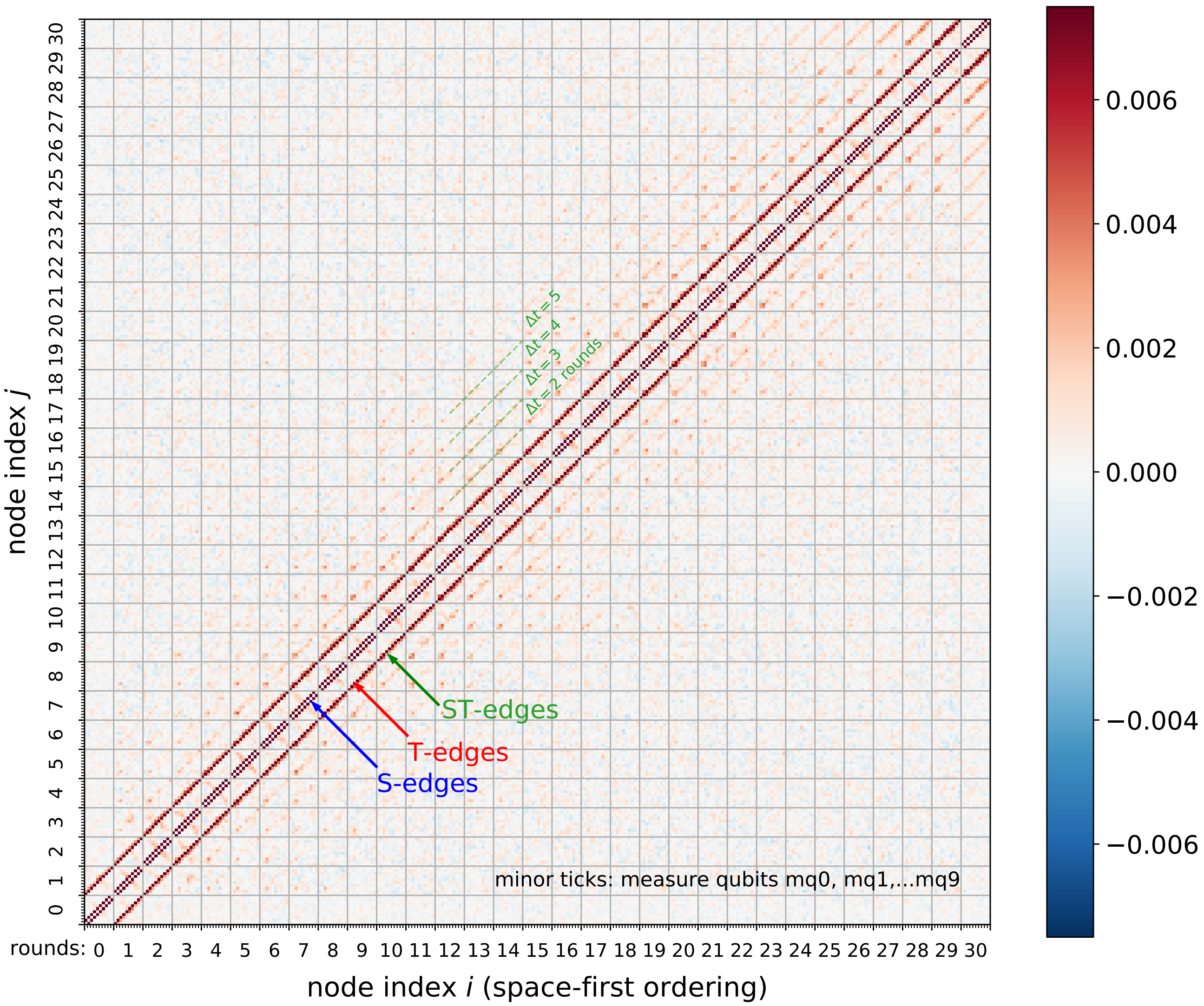

FIG. S21. **Matrix p_{ij} in space-first node ordering.** The figure shows the same data as in Fig. S20, but with the nodes ordered in the "space-first" fashion of Eqn. (8). Each axis contains $N_\mathrm{r} + 1 = 31$ blocks with $N_\mathrm{mq} = 10$ points (ticks) within each block. The lines for S, T, and ST edges are shifted from the main diagonal by 1, 31, and 32 pixels, respectively. Short dashed lines correspond to 2T, 3T, ... edges, which connect nodes separated by $\Delta t = 2, 3, \ldots$ rounds. The well-visible diagonal stripes indicate the presence of long-time correlations in detection events lasting for over 5 rounds.

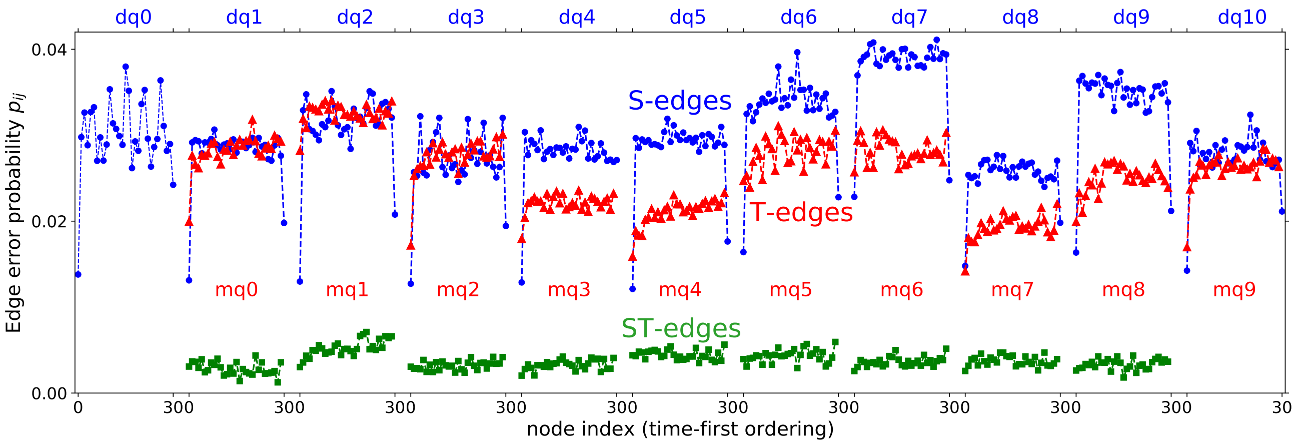

FIG. S22. **S, T, and ST errors.** The plot shows the error probabilities p_{ij} for S (spacelike), T (timelike), and ST (spacetime-like) edges for the data in Fig. S20 (phase-flip code, 10+11 qubits, 30 rounds). For S-edges (blue symbols) the corresponding data qubits dq0–dq10 are indicated at the top, 31 points within each block correspond to rounds. The S-edge probabilities for boundary data qubits dq0 and dq10 are calculated using Eqs. (14)–(16). For T-edges (red symbols), the corresponding measure qubits mq0–mq9 are indicated below the red symbols, each block contains 30 points. ST-edges (green symbols) are positioned in the same way as S-edges (without boundaries), with 30 points per block. Lines are a guide for the eye.

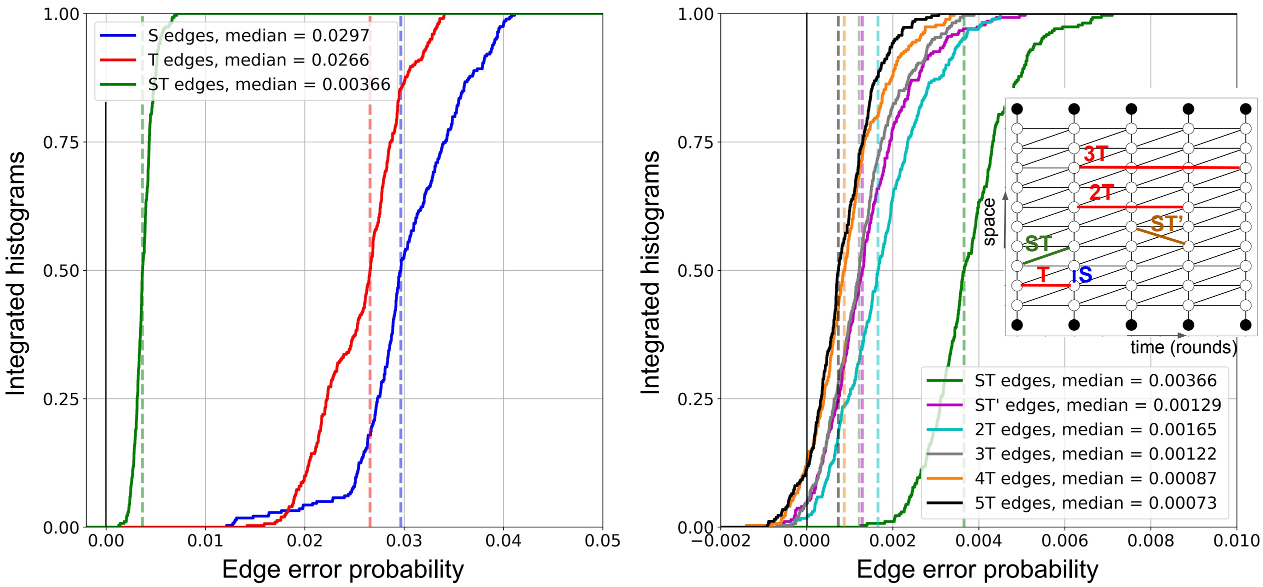

FIG. S23. **Integrated histograms of edge error probabilities.** Left panel: Integrated histograms for the error probabilities p_{ij} of the conventional edges: S (blue line), T (red line), and ST (green line). Median values are indicated by vertical dashed lines and shown in the legend. Right panel: Integrated histograms for ST edges and several unconventional edge types: other-direction spacetimelike edges ST′ and long timelike edges 2T, 3T, 4T, and 5T spanning 2, 3, 4, and 5 rounds. The inset illustrates the edge types on the error graph. Median values (dashed lines) are listed in the legend.

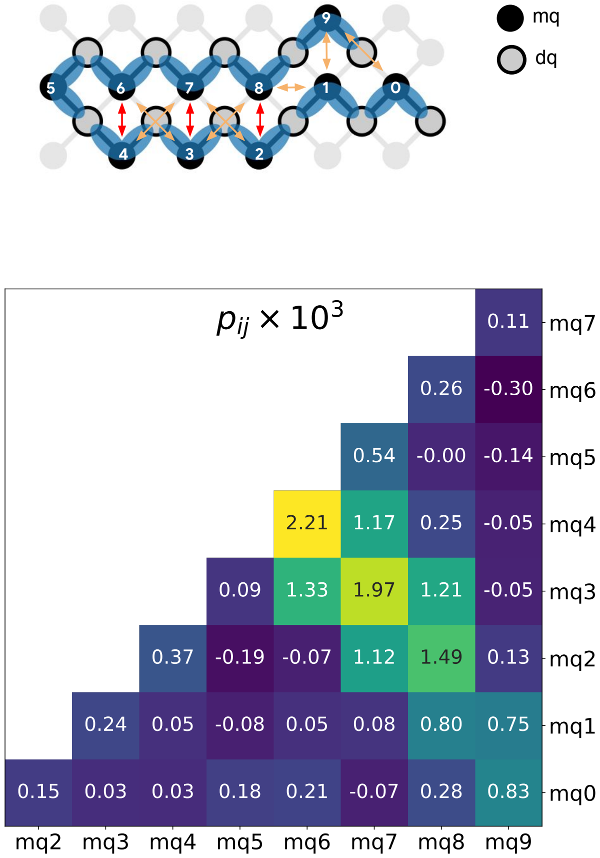

FIG. S24. **Crosstalk error probabilities**. Top panel: Layout of 10 measure qubits (black circles with integer labels) and 11 data qubits (gray-filled circles) on the Sycamore device. Arrows indicate the pairs of measure qubits that exhibit stronger (red arrows) and weaker (orange arrows) detection-event correlations due to crosstalk. Bottom panel: Effective crosstalk probabilities between pairs of measure qubits (except for nearest neighbors). We show the values of $p_{ij} \times 10^3$ for same-round p_{ij} elements averaged over rounds. Cells are colored according to the values: yellow and green indicate a significant crosstalk, blue indicates statistical noise. The biggest crosstalk of 2.2×10^{-3} is between mq4 and mq6 (leftmost arrow in the top panel).

X. COMPARISON OF EDGE WEIGHTING METHODS FOR MATCHING

To decode the error detections obtained in the experiment, we use a minimum weight perfect matching algorithm to determine which physical errors were most likely given the observed directions. A key component of this algorithm is the weighting of the edges in the error graph which correspond to the expected correlated probabilities of pairs of nodes. The weight of a particular edge (W) and the expected probability for that edge (p) are related by

$$W = -\log p \tag{27}$$

which satisfies the property that adding the weights of two edges corresponds to multiplying their probabilities. We considered four candidate strategies for determining expected edge probabilities and weights:

1. **Uniform weighting** - assume that all edges in the matching graph are equally likely
2. **Bootstrapping** - Run matching on a training dataset with uniform weights, then for a given edge, count the number of times it was matched and divide by the number of total experiments to compute the expected probability for future matches.
3. **Node correlations** (p_{ij}) - Use the node correlation technique described in Section IX to determine the correlated probabilities for edges from a training dataset.
4. **First principles** - From the measured gate, measurement, and reset error probabilities, compute the edge probabilities by propagating possible errors through the circuit.

For methods 2 and 3, we use the data at 50 rounds to determine the matching weights for all other datasets. While these methods can in general produce a unique weight for each edge in the 50 round graph, we average together all rounds so that the edge weights used during matching are uniform in time. Phase flip and bit flip edge weights, as well as weights for each of the smaller subsampled codes, are determined separately.

In Tab. S6, we show the fitted values of Λ using the different weighting methods, for both the bit and phase flip codes. To within the uncertainty from fitting, we find that methods 2, 3, 4 all give the same result for Λ_x and Λ_z, while uniform weighting reduces Λ_x to 2.7 and Λ_z to 2.5. The primary effect of the more sophisticated weighting methods is to increase the weights of spacetime edges relative to spacelike and timelike edges.

Other potential techniques for decoding detections include maximum likelihood [18] and neural networks [19, 20]. The efficacy of these other methods compared to minimum weight perfect matching is currently under investigation.

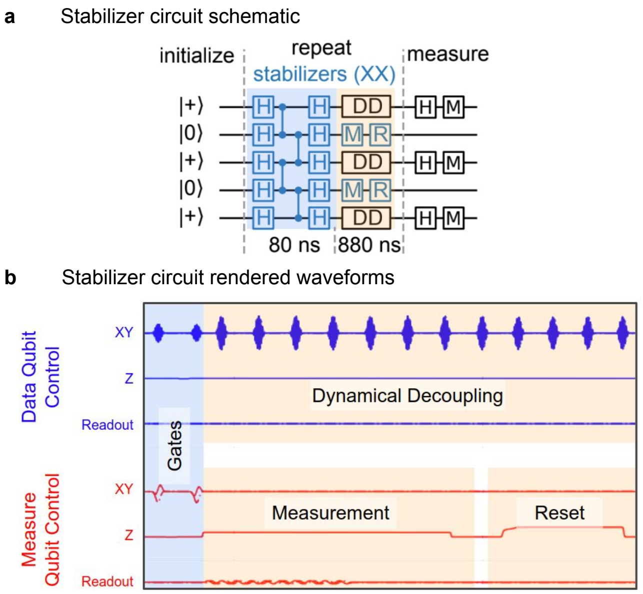

FIG. S25. **Stabilizer Circuit. a,** Circuit schematic representation of the stabilizer circuit. Layers of single qubit and two qubit gates highlighted in blue. Measurement, reset, and dynamical decoupling operations highlighted in yellow to correspond to the waveforms in **b,** Rendered waveforms to show that the majority of the time spent during the stabilizer is during the measurement and reset operations. Lines represent microwave control (XY), flux control (Z), and readout for the stabilizer circuit for one data qubit (blue) and one measure qubit (red).

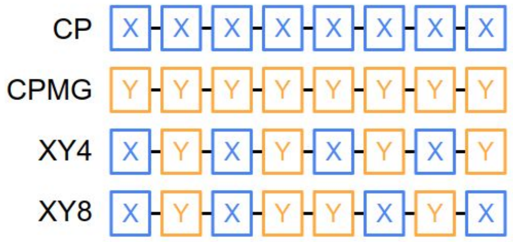

FIG. S26. **Dynamical Decoupling Sequences.** The four multi-pulse sequences used during the measurement and reset portions of the stabilizer circuit. Each sequence has the same total idle time and executes the same number of gates. The distinction between these four sequences during the execution of the circuit is only the phase of the microwave pulses, a technique used to compensate for cumulative pulse errors.

XI. DYNAMICAL DECOUPLING OF DATA QUBITS

The measurement and reset operations take 880 ns to complete and account for approximately 92% of the time spent for the duration of the phase flip code (see Fig. S25). Leaving data qubits to idle during these operations, we undergo energy relaxation processes in addition to dephasing processes, accounting for a large portion of the total error budget. The process of measurement

TABLE S6. Error suppression factors (Λ_x, Λ_z for phase and bit flip) and multiplicative constants (C_x and C_z) fit to logical error rates vs code distance (Eqn. 1 of the main text) for the four different edge weighting methods.

Weighting method	C_x	λ_x	C_z	λ_z
Uniform	0.056 ± 0.005	2.79 ± 0.056	0.066 ± 0.007	2.75 ± 0.06
Bootstrapping	0.068 ± 0.008	3.18 ± 0.08	0.078 ± 0.01	3.01 ± 0.09
Correlation (p_{ij})	0.067 ± 0.008	3.18 ± 0.08	0.077 ± 0.011	3.01 ± 0.09
First principles	0.067 ± 0.007	3.17 ± 0.08	0.0756 ± 0.011	2.99 ± 0.09

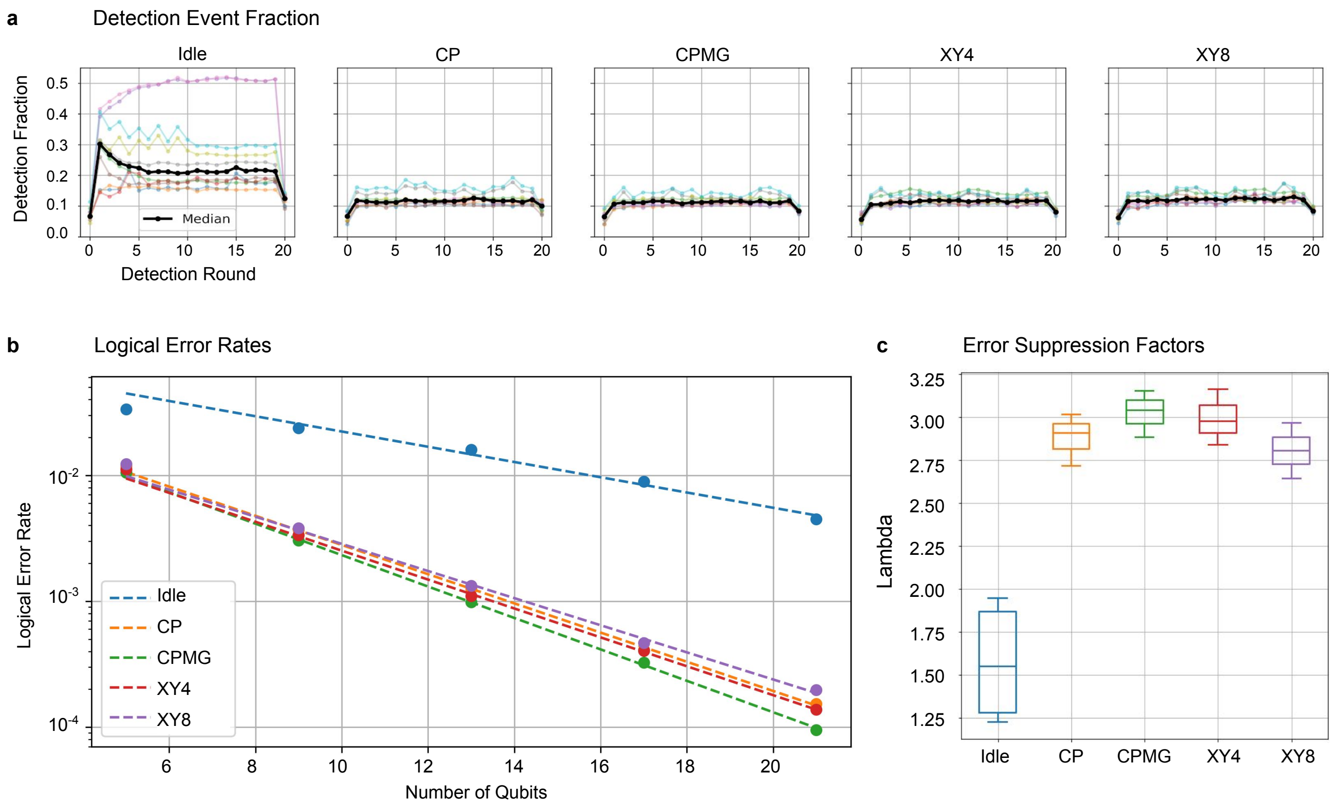

FIG. S27. **Benchmarking phase flip performance with and without dynamical decoupling.** b Detection event fractions vs qubit and round for each of the data qubit Idle, CP, CPMG, XY4, and XY8 operations during measure qubit readout and reset. Median detection event fraction by round plotted in black. **b,** Logical error rate vs number of qubits, showing exponential suppression of error rate in all cases. **c,** Boxplot of extracted error suppression factors (Λ) from fits like those shown in **b,** for five iterations of the experiment for each decoupling scheme. Overall, we see an 1.7x increase in for all decoupling schemes. The performance between the various decoupling schemes is comparable.

and reset on the measure qubits introduces additional avenues of error including measurement-induced dephasing from photon crosstalk between readout resonators [21], as well as frequency detuning errors incurred from any flux crosstalk between qubits. While energy relaxation is irreversible and cannot be mitigated here, dephasing can be mitigated using dynamical-decoupling techniques. We employ multi-pulse sequences developed within the field of NMR which have been shown to mitigate low-frequency noise in superconducting qubits [22]: Carr-Purcell (CP) [23], Car-Purcell-Meiboom-Gill (CPMG) [24], XY4, and XY8 [25].

With independent phase coherence measurements, we verified that we were able to effectively decouple the qubits from the noise sources listed above. Using CPMG, we verified independently via phase coherence measurements with and without adversarial readout tones, as well as with and without large frequency excursions on neighboring qubits, that we are able to effectively decouple away the intrinsic low-frequency noise, measurement-induced dephasing on the data qubits caused by crosstalk from measure, as well as any flux crosstalk effects. We then evaluated the performance of each dynamical decoupling protocol within the context of the repetition code. For all of the decoupling sequences, we fix the time between pulses such that every sequence has the same total

idle time and executes the same number of gates (see Fig. S26). The fixed idle time was set such that each sequence performed eight gates. Using decoupling, we see an $\sim 1.7\times$ increase in the error suppression factor, Λ (Fig. S27). To compare the performance of the different decoupling schemes, the experiment was run and analyzed a total of five times for each of the schemes (Idle, CP, CPMG, XY4, and XY8). The performance between schemes was comparable with the CPMG and XY4 sequences slightly outperforming the CP and XY8 sequences.

XII. QUBIT FREQUENCY OPTIMIZATION

Our processor employs frequency-tunable qubits [1]. Quantum logic gates are executed at two distinct types of frequencies: idle and interaction frequencies, which are collectively referred to as gate frequencies. Qubits idle and execute single-qubit gates at their respective idle frequencies. Neighboring qubit-pairs execute CZ gates at their respective interaction frequencies. All gate frequencies are explicitly or implicitly interdependent due to engineered interactions and/or crosstalk according to the repetition code circuit and its mapping onto our processor. Since many error mechanisms are frequency dependent, we can mitigate errors by constructing and optimizing an error model with respect to gate frequencies.

To construct an error model, we combine error contributions from Z pulse-distortion, relaxation, dephasing, and qubit crosstalk. The Z pulse-distortion model penalizes CZ gates for large frequency excursions. The relaxation and dephasing models penalize SQ and CZ gates for approaching relaxation and dephasing hotspots, while incorporating coupler physics, qubit hybridization, state-dependent transitions, and hardware-accurate frequency trajectories. Finally, the qubit-crosstalk model penalizes for frequency collisions between nearest-neighbor (NN) and diagonal next-nearest-neighbor (NNN) qubits, while incorporating qubit hybridization and the mapping of the repetition code circuit onto our processor. These constituent models are determined via theory and/or experiment, consolidated, and then trained to be predictive of experimentally measured error benchmarks via machine learning.

To determine a frequency configuration that mitigates error, we optimize the error model with respect to gate frequencies. Optimization is complex since the error model spans 41 frequency variables, is non-convex, and time-dependent [26]. Furthermore, since each frequency variable is constrained to $\sim 10^2$ values by the control hardware and qubit-circuit parameters, the optimization search space is $\sim 2^{272}$, which significantly exceeds the Hilbert-space dimension 2^{21}. Given the optimization complexity, exhaustive search is intractable and global optimization is too slow and inefficient. To quickly and efficiently find locally optimal gate-frequency configurations and maintain them in the presence of drift, we use our Snake optimizer [27].

To illustrate the performance of our error mitigation strategy, we conduct a qubit-crosstalk mitigation experiment (see Fig. S28). In this experiment, we first optimize our processor employing one of three qubit-crosstalk mitigation strategies. We then calibrate the processor and run the bit-flip repetition code. The three mitigation strategies are labelled “none”, “partial”, and “full”, according to the expected degree of crosstalk protection. In the “none” strategy, we do not penalize for crosstalk. In the “partial” strategy, we penalize for crosstalk according to the cross-entropy benchmarking (XEB) circuit [1], which we often use in calibration. Although XEB and the repetition code have different circuits and serve different purposes, their respective circuits have similar gate patterns (see Fig. S25 of Ref. [1]). Because of this similarity, penalizing for crosstalk according to XEB should also offer partial crosstalk protection for the repetition code. Finally, in the “full” strategy, we penalize for crosstalk according to the repetition code circuit that we run.

To quantify the efficacy of the three mitigation strategies, we inspect bit-flip repetition-code detection event fractions (DEF). We see that by increasing the degree of crosstalk mitigation from “none” to “partial” to “full”, the median DEF is reduced by 33% and 7%, respectively. Furthermore, the DEF standard-deviation is reduced by 82% and 51%, respectively. In total, this amounts to a 38% reduction in median DEF and a 91% reduction in the DEF standard-deviation, representing a significant performance boost. We delegate error mitigation data for other error mechanisms to a future publication.

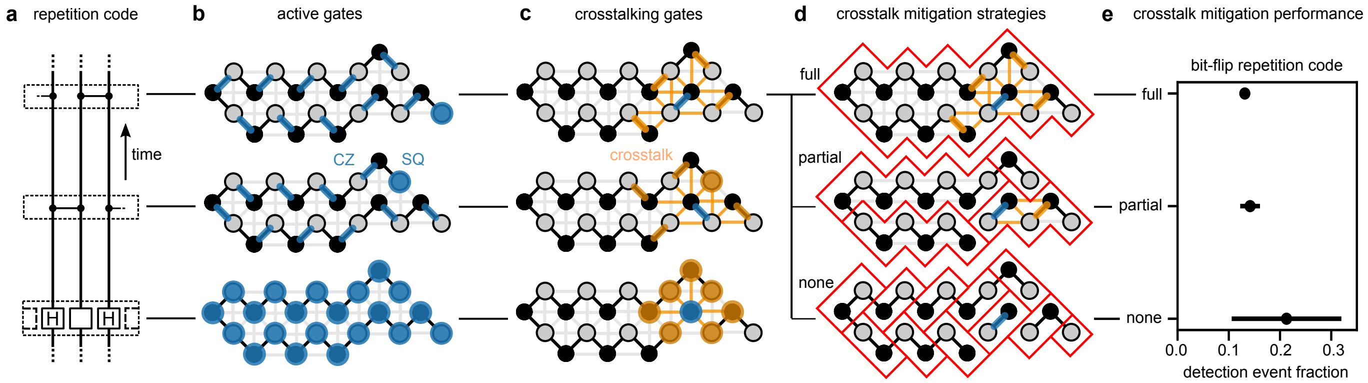

FIG. S28. **Qubit-crosstalk mitigation.** **a,** The repetition code, with three distinct temporal slices indicated by dashed boxes. The empty boxes in the lowest temporal slice are either H or I depending on whether we run the bit- or phase-flip code. **b,** Simultaneously active SQ (H or I) and CZ gates (blue nodes and edges, respectively) at each temporal slice. The geometry of active gates is determined by the repetition code circuit and its mapping onto our processor. Simultaneously active gates can crosstalk due to parasitic interactions between NN and NNN qubits. **c,** Crosstalking SQ and CZ gates (orange nodes and edges, respectively) for one active SQ or CZ (blue nodes and edges, respectively) gate at each temporal slice. We mitigate crosstalk and other error mechanisms by constructing and optimizing an error model with respect to gate-frequencies. **d,** Three crosstalk mitigation strategies illustrated for one active CZ gate in the upper temporal slice in **a** - **c**. The strategies are labelled “full”, “partial”, and “none”, according to the degree of expected crosstalk protection. Each strategy can be characterized by domains (red) in which crosstalk is penalized. **e,** Bit-flip repetition code benchmarks for each mitigation strategy. The points and error bars represent the DEF median and standard-deviation, respectively. By increasing the mitigation strength from “none” to “full”, the DEF median and standard-deviation are reduced by 38% and 91%, respectively.

XIII. OVERVIEW OF ERROR CORRECTION EXPERIMENTS

In Table S7, we list experimental implementations of quantum error correction as a reference.

TABLE S7. Various error correction and error detection experiments. Experiments using "classical" codes (i.e. codes that only detect one type of error e.g. only phase flips or only bit flips) use classical $[n, k, d]$ code notation instead of quantum $[[n, k, d]]$ code notation. Entries with an N/A are experiments related to embedding error correction into the physical qubits as opposed to layering the error correction on top of the physical qubits. Note that there is, as of yet, no experiment exploring a range of rounds and a range of code distances using a non-classical code.

Paper	Year	Code name	[[#data,#logical,distance]]	Physical qubits	Rounds	Physical qubit type
[28]	1998	Repetition Code	[3,1,3]	3	single shot	NMR
[29]	2001	Perfect Code	[[5,1,3]]	5	single shot	NMR
[30]	2011	Repetition Code	[3,1,3]	3	3	Ion trap
[31]	2011	Repetition Code	[3,1,3]	3	2	NMR
[32]	2011	Repetition Code	[3,1,3]	3	single shot	NMR
[33]	2012	Repetition Code	[3,1,3]	3	single shot	Superconducting
[34]	2012	Perfect Code	[[5,1,3]]	5	single shot	NMR
[35]	2014	Surface Code	[[4,1,2]]	4	single shot	Photons
[9]	2014	Repetition Code	[3,1,3]-[5,1,5]	9	8	Superconducting
[36]	2014	Color Code	[[7,1,3]]	7	single shot	Ion trap
[37]	2014	Repetition Code	[3,1,3]	4	single shot	NV center
[38]	2015	Repetition Code	[3,1,3]	5	single shot	Superconducting
[39]	2015	Bell State	[[2,0,2]]	4	single shot	Superconducting
[40]	2016	Repetition Code	[3,1,3]	4	1-3	Superconducting
[41]	2016	Cat States	N/A	1	1-6	3D cavity
[42]	2017	Color Code	[[4,2,2]]	5	single shot	Superconducting
[43]	2017	Color Code	[[4,2,2]]	5	single shot	Ion trap
[44]	2017	Cat States	N/A	1	N/A	superconducting
[45]	2018	Repetition Code	[3,1,3]-[8,1,8]	15	single shot	Superconducting
[46]	2018	Color Code	[[4,2,2]]	5	single shot	superconducting
[47]	2019	Bell State	[[2,0,2]]	3	1-12	Superconducting
[48]	2019	Perfect Code	[[5,1,3]]	5	single shot	Superconducting
[49]	2019	Binomial Bosonic States	N/A	1	1-19	3D cavity
[50]	2019	Color Code	[[4,2,2]]	4	single shot	superconducting
[51]	2020	Repetition Code	[3,1,3]-[22,1,22]	5-43	single shot	Superconducting
[52]	2020	Cat States	N/A	1	single shot	Superconducting
[6]	2020	Surface Code	[[4,1,2]]	7	1-11	Superconducting
[53]	2020	Bell State	[[2,0,2]]	3	1-26	Superconducting
[54]	2020	Bacon-Shor Code	[[9,1,3]]	15	single shot	Ion trap
[55]	2020	Bacon-Shor Code	[[9,1,3]]	11	single shot	Photons
[56]	2020	GKP States	N/A	1	1-200	3D cavity
This work	2021	Repetition Code	[3,1,3]-[11,1,11], [[4,1,2]]	5-21	1-50	Superconducting

[1] F. Arute, K. Arya, R. Babbush, D. Bacon, J. C. Bardin, R. Barends, R. Biswas, S. Boixo, F. G. Brandao, D. A. Buell, *et al.*, Quantum supremacy using a programmable superconducting processor, Nature **574**, 505 (2019).

[2] M. McEwen *et al.*, Removing leakage-induced correlated errors in superconducting quantum error correction, Submitted (2020).

[3] Z. Chen, J. Kelly, C. Quintana, R. Barends, B. Campbell, Y. Chen, B. Chiaro, A. Dunsworth, A. Fowler, E. Lucero, *et al.*, Measuring and suppressing quantum state leakage in a superconducting qubit, Physical review letters **116**, 020501 (2016).

[4] J. Kelly, R. Barends, A. Fowler, A. Megrant, E. Jeffrey, T. White, D. Sank, J. Mutus, B. Campbell, Y. Chen, *et al.*, Scalable in situ qubit calibration during repetitive error detection, Physical Review A **94**, 032321 (2016).

[5] C. Horsman, A. G. Fowler, S. Devitt, and R. V. Meter, Surface code quantum computing by lattice surgery, New Journal of Physics **14**, 123011 (2012).

[6] C. K. Andersen, A. Remm, S. Lazar, S. Krinner, N. Lacroix, G. J. Norris, M. Gabureac, C. Eichler, and A. Wallraff, Repeated quantum error detection in a surface code, Nature Physics **16**, 875 (2020).

[7] A. Y. Kitaev, Fault-tolerant quantum computation by anyons, Annals of Physics **303**, 2 (2003).

[8] A. G. Fowler and C. Gidney, Low overhead quantum computation using lattice surgery (2019), arXiv:1808.06709 [quant-ph].

[9] J. Kelly, R. Barends, A. G. Fowler, A. Megrant, E. Jeffrey, T. C. White, D. Sank, J. Y. Mutus, B. Campbell, Y. Chen, *et al.*, State preservation by repetitive error detection in a superconducting quantum circuit, Nature **519**, 66 (2015).

[10] D. Gottesman, *Stabilizer Codes and Quantum Error Correction*, Ph.D. thesis, Pasadena, CA (1997).

[11] S. Bravyi and A. Kitaev, Universal quantum computation with ideal clifford gates and noisy ancillas, Phys. Rev. A **71**, 022316 (2005).

[12] S. Anders and H. J. Briegel, Fast simulation of stabilizer circuits using a graph-state representation, Phys. Rev. A **73**, 022334 (2006).

[13] R. Barends, C. M. Quintana, A. G. Petukhov, Y. Chen, D. Kafri, K. Kechedzhi, R. Collins, O. Naaman, S. Boixo, F. Arute, K. Arya, D. Buell, B. Burkett, Z. Chen, B. Chiaro, A. Dunsworth, B. Foxen, A. Fowler, C. Gidney, M. Giustina, R. Graff, T. Huang, E. Jeffrey, J. Kelly, P. V. Klimov, F. Kostritsa, D. Landhuis, E. Lucero, M. McEwen, A. Megrant, X. Mi, J. Mutus, M. Neeley, C. Neill, E. Ostby, P. Roushan, D. Sank, K. J. Satzinger, A. Vainsencher, T. White, J. Yao, P. Yeh, A. Zalcman, H. Neven, V. N. Smelyanskiy, and J. M. Martinis, Diabatic gates for frequency-tunable superconducting qubits, Phys. Rev. Lett. **123**, 210501 (2019).

[14] A. G. Fowler, A. C. Whiteside, and L. C. Hollenberg, Towards practical classical processing for the surface code, Physical review letters **108**, 180501 (2012).

[15] S. Bravyi, M. Suchara, and A. Vargo, Efficient algorithms for maximum likelihood decoding in the surface code, Phys. Rev. A **90**, 032326 (2014).

[16] A. G. Fowler, Minimum weight perfect matching of fault-tolerant topological quantum error correction in average o (1) parallel time, Quantum Information & Computation **15**, 145 (2015).

[17] S. T. Spitz, B. Tarasinski, C. W. Beenakker, and T. E. O'Brien, Adaptive weight estimator for quantum error correction in a time-dependent environment, Advanced Quantum Technologies **1**, 1800012 (2018).

[18] B. M. Terhal, Quantum error correction for quantum memories, Reviews of Modern Physics **87**, 307 (2015).

[19] Y.-H. Liu and D. Poulin, Neural belief-propagation decoders for quantum error-correcting codes, Physical review letters **122**, 200501 (2019).

[20] S. Varsamopoulos, B. Criger, and K. Bertels, Decoding small surface codes with feedforward neural networks, Quantum Science and Technology **3**, 015004 (2017).

[21] D. Szombati, A. G. Frieiro, C. Müller, T. Jones, M. Jerger, and A. Fedorov, Quantum rifling: Protecting a qubit from measurement back action, Physical Review Letters **124**, 070401 (2020).

[22] J. Bylander, S. Gustavsson, F. Yan, F. Yoshihara, K. Harrabi, G. Fitch, D. G. Cory, Y. Nakamura, J.-S. Tsai, and W. D. Oliver, Noise spectroscopy through dynamical decoupling with a superconducting flux qubit, Nature Physics **7**, 565 (2011).

[23] H. Y. Carr and E. M. Purcell, Effects of diffusion on free precession in nuclear magnetic resonance experiments, Physical review **94**, 630 (1954).

[24] S. Meiboom and D. Gill, Modified spin-echo method for measuring nuclear relaxation times, Review of scientific instruments **29**, 688 (1958).

[25] T. Gullion, D. B. Baker, and M. S. Conradi, New, compensated carr-purcell sequences, Journal of Magnetic Resonance (1969) **89**, 479 (1990).

[26] P. Klimov, J. Kelly, Z. Chen, M. Neeley, A. Megrant, B. Burkett, R. Barends, K. Arya, B. Chiaro, Y. Chen, *et al.*, Fluctuations of energy-relaxation times in superconducting qubits, Physical review letters **121**, 090502 (2018).

[27] P. V. Klimov, J. Kelly, J. M. Martinis, and H. Neven, The snake optimizer for learning quantum processor control parameters, arXiv preprint arXiv:2006.04594 (2020).

[28] D. G. Cory, M. Price, W. Maas, E. Knill, R. Laflamme, W. H. Zurek, T. F. Havel, and S. S. Somaroo, Experimental quantum error correction, Physical Review Letters **81**, 2152 (1998).

[29] E. Knill, R. Laflamme, R. Martinez, and C. Negrevergne, Benchmarking quantum computers: the five-qubit error correcting code, Physical Review Letters **86**, 5811 (2001).

[30] P. Schindler, J. T. Barreiro, T. Monz, V. Nebendahl, D. Nigg, M. Chwalla, M. Hennrich, and R. Blatt, Experimental repetitive quantum error correction, Science **332**, 1059 (2011).

[31] O. Moussa, J. Baugh, C. A. Ryan, and R. Laflamme, Demonstration of sufficient control for two rounds of quantum error correction in a solid state ensemble quantum information processor, Physical review letters **107**, 160501 (2011).

[32] J. Zhang, D. Gangloff, O. Moussa, and R. Laflamme, Experimental quantum error correction with high fidelity, Physical Review A **84**, 034303 (2011).

[33] M. D. Reed, L. DiCarlo, S. E. Nigg, L. Sun, L. Frunzio, S. M. Girvin, and R. J. Schoelkopf, Realization of

three-qubit quantum error correction with superconducting circuits, Nature **482**, 382 (2012).

[34] J. Zhang, R. Laflamme, and D. Suter, Experimental implementation of encoded logical qubit operations in a perfect quantum error correcting code, Physical review letters **109**, 100503 (2012).

[35] B. Bell, D. Herrera-Martí, M. Tame, D. Markham, W. Wadsworth, and J. Rarity, Experimental demonstration of a graph state quantum error-correction code, Nature communications **5**, 1 (2014).

[36] D. Nigg, M. Mueller, E. A. Martinez, P. Schindler, M. Hennrich, T. Monz, M. A. Martin-Delgado, and R. Blatt, Quantum computations on a topologically encoded qubit, Science **345**, 302 (2014).

[37] G. Waldherr, Y. Wang, S. Zaiser, M. Jamali, T. Schulte-Herbrüggen, H. Abe, T. Ohshima, J. Isoya, J. Du, P. Neumann, *et al.*, Quantum error correction in a solid-state hybrid spin register, Nature **506**, 204 (2014).

[38] D. Riste, S. Poletto, M.-Z. Huang, A. Bruno, V. Vesterinen, O.-P. Saira, and L. DiCarlo, Detecting bit-flip errors in a logical qubit using stabilizer measurements, Nature communications **6**, 1 (2015).

[39] A. D. Córcoles, E. Magesan, S. J. Srinivasan, A. W. Cross, M. Steffen, J. M. Gambetta, and J. M. Chow, Demonstration of a quantum error detection code using a square lattice of four superconducting qubits, Nature communications **6**, 1 (2015).

[40] J. Cramer, N. Kalb, M. A. Rol, B. Hensen, M. S. Blok, M. Markham, D. J. Twitchen, R. Hanson, and T. H. Taminiau, Repeated quantum error correction on a continuously encoded qubit by real-time feedback, Nature communications **7**, 1 (2016).

[41] N. Ofek, A. Petrenko, R. Heeres, P. Reinhold, Z. Leghtas, B. Vlastakis, Y. Liu, L. Frunzio, S. Girvin, L. Jiang, *et al.*, Extending the lifetime of a quantum bit with error correction in superconducting circuits, Nature **536**, 441 (2016).

[42] M. Takita, A. W. Cross, A. Córcoles, J. M. Chow, and J. M. Gambetta, Experimental demonstration of fault-tolerant state preparation with superconducting qubits, Physical review letters **119**, 180501 (2017).

[43] N. M. Linke, M. Gutierrez, K. A. Landsman, C. Figgatt, S. Debnath, K. R. Brown, and C. Monroe, Fault-tolerant quantum error detection, Science advances **3**, e1701074 (2017).

[44] R. W. Heeres, P. Reinhold, N. Ofek, L. Frunzio, L. Jiang, M. H. Devoret, and R. J. Schoelkopf, Implementing a universal gate set on a logical qubit encoded in an oscillator, Nature communications **8**, 1 (2017).

[45] J. R. Wootton and D. Loss, Repetition code of 15 qubits, Physical Review A **97**, 052313 (2018).

[46] D. Willsch, M. Willsch, F. Jin, H. De Raedt, and K. Michielsen, Testing quantum fault tolerance on small systems, Physical Review A **98**, 052348 (2018).

[47] C. K. Andersen, A. Remm, S. Lazar, S. Krinner, J. Heinsoo, J.-C. Besse, M. Gabureac, A. Wallraff, and C. Eichler, Entanglement stabilization using ancilla-based parity detection and real-time feedback in superconducting circuits, npj Quantum Information **5**, 1 (2019).

[48] M. Gong, X. Yuan, S. Wang, Y. Wu, Y. Zhao, C. Zha, S. Li, Z. Zhang, Q. Zhao, Y. Liu, *et al.*, Experimental verification of five-qubit quantum error correction with superconducting qubits, arXiv preprint arXiv:1907.04507 (2019).

[49] L. Hu, Y. Ma, W. Cai, X. Mu, Y. Xu, W. Wang, Y. Wu, H. Wang, Y. Song, C.-L. Zou, *et al.*, Quantum error correction and universal gate set operation on a binomial bosonic logical qubit, Nature Physics **15**, 503 (2019).

[50] R. Harper and S. T. Flammia, Fault-tolerant logical gates in the ibm quantum experience, Physical review letters **122**, 080504 (2019).

[51] J. R. Wootton, Benchmarking near-term devices with quantum error correction, arXiv preprint arXiv:2004.11037 (2020).

[52] R. Lescanne, M. Villiers, T. Peronnin, A. Sarlette, M. Delbecq, B. Huard, T. Kontos, M. Mirrahimi, and Z. Leghtas, Exponential suppression of bit-flips in a qubit encoded in an oscillator, Nature Physics **16**, 509 (2020).

[53] C. Bultink, T. O'Brien, R. Vollmer, N. Muthusubramanian, M. Beekman, M. Rol, X. Fu, B. Tarasinski, V. Ostroukh, B. Varbanov, *et al.*, Protecting quantum entanglement from leakage and qubit errors via repetitive parity measurements, Science advances **6**, eaay3050 (2020).

[54] L. Egan, D. M. Debroy, C. Noel, A. Risinger, D. Zhu, D. Biswas, M. Newman, M. Li, K. R. Brown, M. Cetina, *et al.*, Fault-tolerant operation of a quantum error-correction code, arXiv preprint arXiv:2009.11482 (2020).

[55] Y.-H. Luo, M.-C. Chen, M. Erhard, H.-S. Zhong, D. Wu, H.-Y. Tang, Q. Zhao, X.-L. Wang, K. Fujii, L. Li, *et al.*, Quantum teleportation of physical qubits into logical code-spaces, arXiv preprint arXiv:2009.06242 (2020).

[56] P. Campagne-Ibarcq, A. Eickbusch, S. Touzard, E. Zalys-Geller, N. Frattini, V. Sivak, P. Reinhold, S. Puri, S. Shankar, R. Schoelkopf, *et al.*, Quantum error correction of a qubit encoded in grid states of an oscillator, Nature **584**, 368 (2020).
